# Technical feasibility, sustainability and technoeconomic analysis of non-wood fibre integration in paper-based flexible packaging: a case study of India

**DOI:** 10.1039/d6su00291a

**Published:** 2026-11-09

**Authors:** Aaron E. Brown, Paulina Gordina, Meiju Sinkkonen, Karim Ismail, Tekla Tammelin, Andrew Ross, Koon-Yang Lee, John Blacker, Keeran Ward

**Affiliations:** a School of Chemical and Process Engineering, University of Leeds Woodhouse Lane Leeds LS2 9JT UK k.r.ward@leeds.ac.uk; b Department of Aeronautics, Imperial College London South Kensington Campus London SW7 2AZ UK; c VTT Technical Research Centre of Finland Ltd PO Box 1603 FI-40101 Jyväskylä Finland; d VTT Technical Research Centre of Finland Ltd PO Box 1000 FI-02044 Espoo Finland; e Institute of Process Research and Development, School of Chemistry, University of Leeds Leeds LS2 9JT UK

## Abstract

The environmental persistence of flexible plastics has driven a shift towards the development of paper-based alternatives. Integrating non-wood fibres into paper production is receiving increasing interest due to perceived environmental benefits and cost reduction compared to papers produced from virgin wood sources. This study investigates how integrating non-wood fibres impacts the physical properties, production costs and environmental impacts of paper produced in India, which represents a market with high volumes of products sold in flexible packaging. Papers in India are largely produced from combinations of softwood and hardwood pulps with a high dependence on softwood imports. Bagasse, bamboo and old corrugated cardboard were utilised to substitute 20% of virgin wood fibres; softwood, hardwood or both. Paper hand sheets were produced using TAPPI T205 sp-02 and key performance indicators including tensile index, porosity, air permeability and surface roughness, were characterised. Substituting imported softwood with locally-sourced non-wood fibres relates to a production cost of $761–$770 USD per ton and a global warming potential (GWP) between 1.88 and 1.93 kg CO_2_ eq. per kg paper, representing an 11–12% reduction in cost and 27–29% reduction in GWP compared to the virgin wood fibre benchmark. Cost and GWP savings are driven by reduced transport impacts to import the softwood and establishing more localised supply chains. Non-wood fibre inclusion generally maintains or improves the physical properties of papers, compared to the virgin wood benchmark. Hand sheets produced using bagasse as a substitute for softwood displayed a tensile index of 28.9 N m g^−1^ and a Gurley time of 1.0 s/10 mL, a 29% and 43% increase compared to the virgin wood benchmark, respectively. Porosity and surface roughness values remained similar to the control. Inclusion of cellulose nanofibre and cationic starch additives further improved hand sheet properties. Overall, the integration of localised non-wood fibres into an uncoated paper substrate for flexible packaging applications offers potential economic and environmental benefits within India, without significant compromise to the physical properties of papers.

## Abstract 2

Hollistic assessment of the physical properties, production costs and environmental impacts of paper hand sheets produced from wood and non-wood blends.

## Graphical abstract

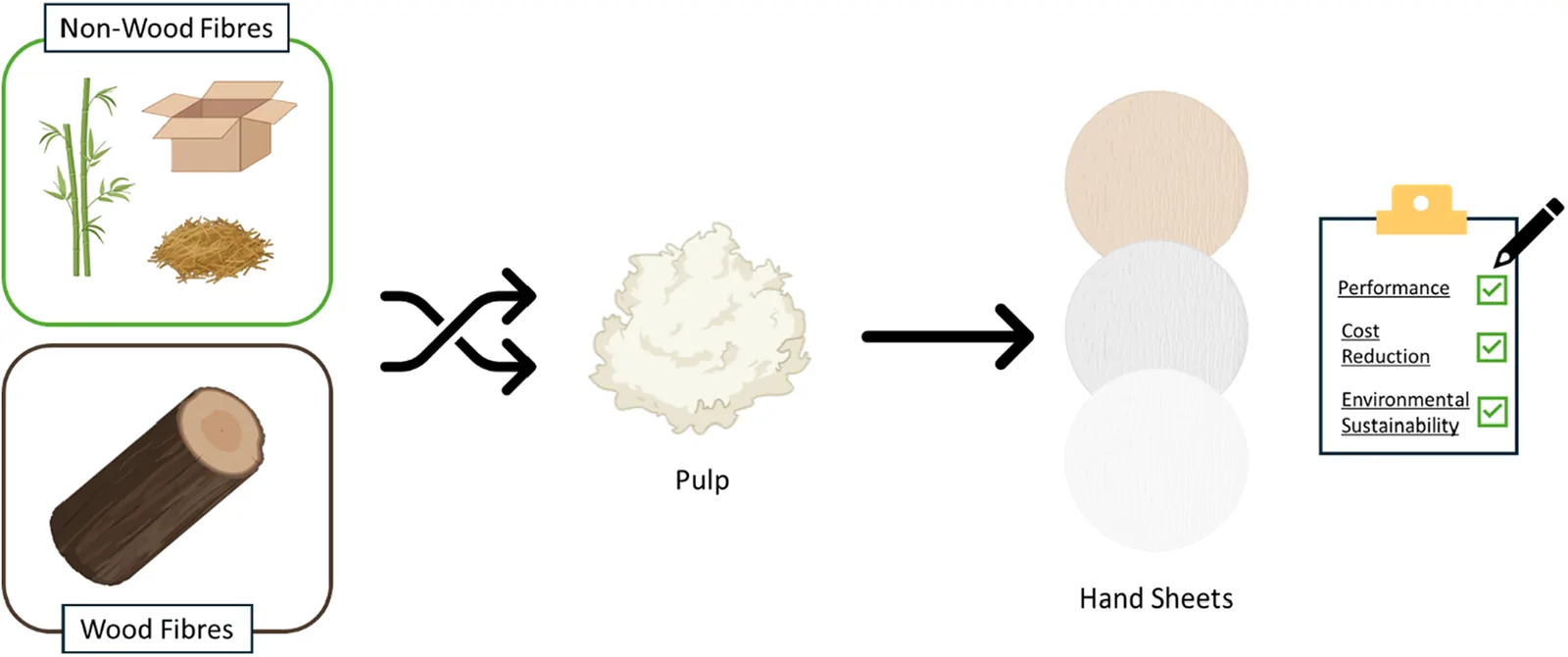

**Sustainability spotlight**

This work advances sustainable packaging by exploring the integration of non-wood fibres into uncoated paper substrates for flexible packaging applications, as an alternative to conventional wood-based papers. It takes a systems-level perspective to evaluate the role of locally available biomass in enabling more resource-efficient and circular production pathways, particularly in rapidly developing economies such as India. By supporting the transition away from fossil-based flexible packaging and reducing reliance on imported raw materials, the study contributes to more resilient and sustainable supply chains. This work aligns with SDG 12 (Responsible Consumption and Production), SDG 13 (Climate Action), and SDG 9 (Industry, Innovation and Infrastructure), promoting low-impact materials innovation and sustainable industrial development.

## Introduction

Plastics have become a part of everyday life, driven by their effectiveness as a packaging material: lightweight, durable, non-reactive, inexpensive, and providing an effective barrier against moisture and oxygen.^1^ However, increased reliance on plastics has resulted in a significant global environmental challenge. The use of plastics is projected to increase nearly 3-fold between 2019 (460 Mt per year) and 2060 (1321 Mt per year), driven mainly by increasing growth in developing economies.^2^ By 2060, plastic usage for packaging materials alone is projected to reach 381 Mt per year; representing the largest sector of global plastic usage, at 31% of overall usage.^2^ It is estimated that just 9% of global total plastic ever produced has undergone effective recycling.^3^ India represents a particularly challenging market for overcoming the issue of plastic waste from packaging materials. In 2019, India produced around 17 Mt of plastic, 59% of which was linked to packaging materials.^4^ The rates of effective plastic recycling in India are low, approximately 15%, with the remaining waste plastic directed towards landfill or incineration.^4^ Fast moving consumer goods (FMCG) companies represent the fourth largest sector of the Indian economy, providing a range of consumer products, including household cleaning, personal care, hygiene and food items.^5^ In India, FMCG products are frequently sold in flexible plastic packaging at lower product doses and in smaller pack sizes compared to more developed countries, to cater towards economically disadvantaged markets.^5^ However, the consequence of selling larger volumes of smaller unit packs is an increase in the number of pack units released into the environment after the product is used, particularly as formal recycling infrastructure is not well-established.^4^ It is estimated that 2.94 billion sachets are discarded weekly in India.^6^ Currently, there are two main formats of flexible plastic packaging: multi-material and mono-material.^4^ Multi-material flexible packaging formats are composed of multiple different layers of polymers (often polypropylene or polyethylene) or metallised layers and are classed as non-recyclable due to the complexities of separating the different layers. Mono-material flexibles are composed of a singular polymer type. Although technically recyclable, the recycling rates of mono-material flexible packaging in India are low (12%), due to their low desirability in small-scale waste aggregator (informal waste pickers) systems, typically utilised across India.^4^ Consequently, the post-consumer fate of flexible packaging in India is often landfilled, incinerated or littered to terrestrial or marine environments. Neo *et al.*^7^ compared the environmental impact of mixed plastic waste across different end-of-life scenarios within India. Open incineration resulted in a higher global warming potential (2.75 kg CO_2_ eq. per kg waste plastic), compared to open landfill (0.13 kg CO_2_ eq. per kg waste plastic) and recycling (between −0.92 and −0.94 kg CO_2_ eq. per kg waste plastic). Landfilling was found to be a major contributor towards marine ecosystem toxicity.^7^

FMCG plastic waste has been widely recognised as a significant environmental issue. Over the last few decades there has been increased interest from multiple stakeholders (government, charity organisations, manufacturers and consumers) for FMCG companies to transition towards more sustainable packaging options.^8–10^ Consequently, there is a resurging interest in the utilisation of paper-based packaging materials. These multi-material structures are predominantly composed of paper, but rely on functional barrier coatings to enable functionality such as moisture and gas resistance and heat sealability.^11^

Paper offers the combined advantages of being bio-based, bio-degradable and recyclable,^10^ while also being perceived by consumers as an environmentally friendly option.^8,12,13^ However, perceived improvements in sustainability must align with conclusions from rigorous scientific investigations, applying tools such as life cycle assessment (LCA). To date, there is still significant debate within the scientific community as to whether plastic or paper displays a ‘lower’ environmental impact as a packaging material. Arfelli *et al.*^14^ concluded that a paper-based chocolate bar packaging demonstrated better performance in 6–10 out of 18 environmental impact categories, including global warming potential, fossil resource scarcity and water consumption, compared to a polypropylene based packaging (depending on the LCA scenario). Alternatively, a recent review by Dolci *et al.*^13^ highlighted three studies comparing the environmental impact of paper and plastic carrier bags, all of which concluded that paper bags displayed an overall worse environmental impact than plastic bags. Meng *et al.*^15^ identified similar findings, concluding that paper grocery bags generate three-times more greenhouse gas (GHG) emissions compared to high-density polyethylene (HDPE) bags. The increased emissions from paper were driven by higher raw material usage and release of methane emissions through anaerobic biodegradation during landfilling, whereas plastics remain relatively inert to producing GHG. However, leachate to groundwater can result in other environmental impacts, such as marine ecotoxicity.^7^ There is often a focus on comparing GHG emissions as a measure of environmental impact; however, other impact categories, such as land use occupation often reflect more negatively on paper-based materials, than plastic-based solutions.^14^

There is a recent upsurge in interest in the utilisation of non-wood fibres for paper production.^16,17^ Non-wood fibres encompass a range of different materials, including agri-waste fibres (*e.g.* sugarcane bagasse, rice straw, and wheat straw), purpose-grown crops (*e.g.* bamboo and miscanthus) and recycled fibres (*e.g.* old corrugated cardboard (OCC).^18^ Non-wood fibres are often perceived as a more sustainable alternative to traditional wood-based fibres, due to their widespread availability, rapid growth rates, mitigation of deforestation and lower costs.^18,19^ However, more detailed LCA-based studies^20,21^ discuss a higher environmental impact for non-wood fibres compared to virgin wood counterparts. Forfora *et al.*^21^ reported an 8% increase in global warming potential (GWP) for a tissue product with a fibre furnish of 70% bamboo and 30% hardwood, than a tissue product with a 70% hardwood and 30% softwood composition. Increased GWP emissions were associated with the manufacture of bamboo pulp in China, a location with a carbon-intensive energy mix. Sun *et al.*^20^ reported that the GWP of straw-based pulp was 4.5–7.9 times that of kraft wood pulp due to higher energy and chemical demand of straw-based pulps. The comparison of LCA studies is complex, due to differences in system boundary, scope, supply chain geography and functional unit. In particular, geographical factors such as energy mix, transportation distances and raw material sourcing can substantially alter the environmental performance attributed to a given fibre type, leading to the inconsistent conclusions observed across previous studies. Favero *et al.*^17^ presented a review of LCA studies for the utilisation of non-wood fibres in papers. The review showed that one particular type of non-wood fibre did not provide a definitive environmental advantage over wood-based fibres, with each study being case-specific, creating difficulties in drawing meaningful conclusions.

The aim of this study is to explore the use of non-wood fibres (bagasse, bamboo and recycled fibres) as a replacement for virgin wood fibres within the paper fraction of paper-based flexible packaging for the Indian market. The resulting paper is not intended to act as a direct substitute for plastic packaging, but rather as a high strength substrate requiring subsequent application of barrier coatings to achieve properties such as moisture resistance and sealability. The novelty of this work is that it implements a whole-systems approach to holistically investigate the impacts of integrating non-wood fibres on paper physical properties, environmental impacts and production costs within the Indian market.

## Methodology

### Materials and chemicals

Five pulp samples were utilised as part of this study. Northern softwood bleached kraft pulp and eucalyptus hardwood pulp were purchased from Merck (USA). Unbleached bagasse and bamboo kraft pulps were purchased from the Sustainable and Alternative Fibers Initiative (SAFI), North Carolina State University. Old corrugated cardboard (OCC) was provided by DS Smith (UK).

Cationic starch grade HI-CAT® C483A was purchased from Roquette (UK). Nanocellulose in the form of an aqueous gel of cellulose nanofibers (CNFs) was used in this study. CNFs were produced through the mechanical disintegration of dried birch kraft pulp obtained from a mill in Finland.^22^ The dried birch kraft pulp contained approximately 73% glucose, 23% xylose, 0.2% residual lignin, 0.09% residual extractives, with the remainder composed of mannose and acidic oligomers. The aqueous CNF gel had a solid content of 2.2 wt%, a pH of 5.8 and a conductivity of 117 µS cm^−1^.^22^

### Hand sheet formation

Hand sheets were produced according to TAPPI T 205 sp-02 using a Pulp Disintegrator (PD-10 model, TECHLABSYSTEMS, Lezo, Spain) and Hand Sheet Former (FTH-1 model, TECHLABSYSTEMS, Lezo, Spain), with a target grammage of 60 gsm. Hand sheets were produced with and without the inclusion of additives: 1 wt% cationic starch and 2.5 wt% CNF. However, the retention efficiency of additives was not quantified. Fig. 1 provides an overview of the hand sheet production process. Pulp moisture values were used to calculate the addition of 24 g dry-equivalent pulp to 2 L of water, which was homogenised in the pulp disintegrator for 30 000 revolutions. The residual pulp slurry was diluted from a 1.2 wt% solution, to a 0.3 wt% solution, and 400 mL was added to the hand sheet former to produce a hand sheet. The 400 mL aliquot of pulp suspension contained the equivalent of approximately 1.2 g dry pulp, which formed a hand sheet over a 199 cm^2^ piece of wire mesh, with a target hand sheet density of 60 gsm. Wet hand sheets were partially dried between blotting paper and polished metal plates, using a Carver hot press set at 345 kPa and 20 °C for a period of 5 min and a subsequent 2 min, replacing the blotting paper in between presses. After pressing, the hand sheets were mounted onto a drying rack and conditioned at 23 °C and 50% RH for at least 24 h before characterisation tests were performed. Example images of hand sheets are displayed in Fig. S1 (SI).

**Fig. 1:**
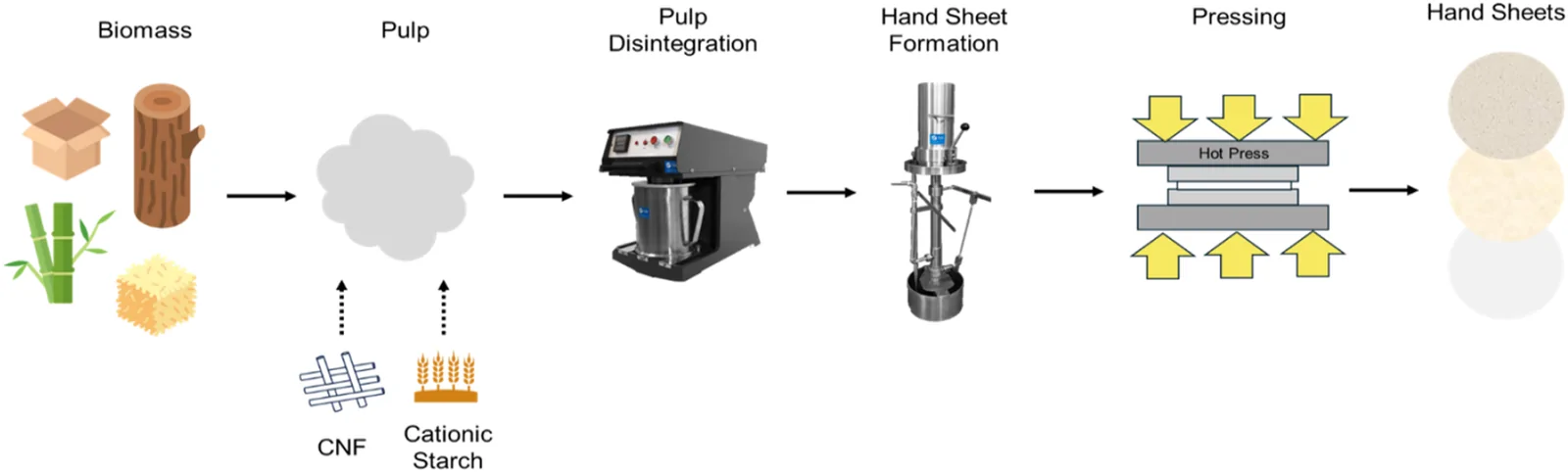
Schematic of hand sheet formation, according to TAPPI T 205 sp-02.

### Sample labelling

Table 1 outlines the different compositions of fibre blends used to produce hand sheets for this study. The business as usual (BAU) fibre furnish was composed of 30% softwood and 70% hardwood, representing a typical composition of virgin wood fibre-based packaging paper commonly used in the Indian market.^23,24^ Using this BAU as a comparative allows us to understand the influence of both fibre furnish and additive inclusion on hand sheet properties without the synergistic effects of wet and dry strength agents. A 20% inclusion level of non-wood fibres was considered, to investigate the impacts on hand sheet properties, compared to the BAU control as a baseline. A 20% non-wood inclusion was selected based on prior work and operational data from Indian paper mills. To investigate the impacts on absolute sustainability and properties of hand sheets, fit-for-purpose in the Indian market, non-wood fibre displacement strategies were considered whereby, non-wood fibres displaced a portion of softwood (SW), hardwood (HW) or a combination of both (SWHW) from the BAU fibre furnish. Bagasse (BAG), bamboo (BAM) and old-corrugated cardboard (OCC) were selected as the non-wood fibres for investigation.

**Table 1:** Composition of hand sheet samples, alongside the sample ID codes^a^

Sample ID	Composition of fibre mix (%)				
	Softwood	Hardwood	Bagasse	Bamboo	OCC
BAU	30	70	0	0	0
BAG-SWHW	24	56	20	0	0
BAG-SW	10	70	20	0	0
BAG-HW	30	50	20	0	0
BAM-SWHW	24	56	0	20	0
BAM-SW	10	70	0	20	0
BAM-HW	30	50	0	20	0
OCC-SWHW	24	56	0	0	20
OCC-SW	10	70	0	0	20
OCC-HW	30	50	0	0	20

a BAU = business as usual control. BAG = bagasse. BAM = bamboo. OCC = old corrugated cardboard. SW = softwood substitution. HW = hardwood substitution. SWHW = both softwood and hardwood substitution.

### Pulp characterisation

Lignocellulosic composition of pulps was determined at an external laboratory (Celignis Analytical) *via* wet-chemistry analysis.^25^ Fibre morphology analysis was measured according to ISO 16065-2 using the L&W Fiber Tester Code 912 Plus measurement system. Both lignocellulosic composition and fibre morphology analyses were conducted in duplicate.

### Hand sheet characterisation

Standard paper characterisation protocols were used to evaluate the performance of each hand sheet. These included: grammage, thickness, porosity, air permeability, tensile index and surface roughness.

### Grammage, thickness and porosity

Hand sheet grammage (*G*) was calculated according to the ratio of mass (*m*) and area (*A*) of each sheet. The thickness (*d*) of hand sheets was measured using a digital micrometer (Moore & Wright Digital Micrometer). Envelope density (*ρ*_e_) was calculated as the ratio between *G* and *d*. The porosity of the hand sheets was then calculated according to eqn (1), assuming a true cellulose density (*ρ*_c_) of 1500 kg m^−3^.^26^1
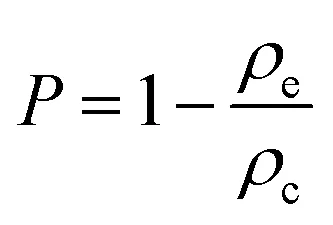


### Air permeability

Air permeability was measured using a Gurley air densometer (Model 4150N, Gurley Precision Instruments). An automatic digital timer (Model 4320, Genuinely Gurley™) was used to measure the time (s) taken for 10 mL of air to pass through a hand sheet. Samples in which air passed through faster than the timer's lower measurable limit were recorded as 0 s. These values represent left-censored values below the detection limit, not true zeros.

### Tensile index

Tensile index (TI) was measured according to ISO 1924-2:2008 using an Instron 34TM-10, with 100 N load cell. TI was calculated according to eqn (2), where *T* represents the tensile strength (N m^−1^) and *G* is the grammage (gsm). A test piece area of 70 mm × 25 mm (*L* × *W*) was produced using a die cutter (Hanatek Instruments, Cutting Die CDC2009001A). A constant rate of elongation of 20 mm min^−1^ was applied until the point of sample break. Tensile index was calculated as follows:2
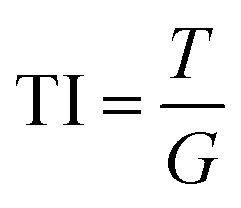


### Surface roughness

Surface area roughness (*S*_a_) of hand sheets was calculated according to ISO 25178-1:2016 using a Sensofar S Neox microscope 3D Optical Profiler with a Nikon TU Plan Fluor EPI 10X-N lens. A test area of 1.75 × 1.32 mm^2^ was measured across a 120 µm *z*-direction with symmetric scan definition measured using a red LED light source (13.0%), gain (40) and gamma (24). Surface roughness was reported as *S*_a_; arithmetical mean height.

### Replicates and statistical analysis

Three independent hand sheets were prepared for each formulation. During characterisation, one measurement was taken per hand sheet for all properties except surface roughness, giving triplicate (*n* = 3) values per formulation. Surface roughness was measured at three positions per sheet (nine measurements per formulation); the three within-sheet measurements were averaged before statistical analysis, providing *n* = 3 values per formulation, maintaining consistency with the other measured properties. Average values are presented alongside error bars representing ± one standard deviation, calculated using MS Excel. Further statistical analyses were performed using IBM SPSS Statistics (Version 29). Differences across all 24 formulations (six reference fibres: BAU, SW, HW, BAG, BAM and OCC; nine blends without additives; and nine blends with additives) were assessed using a one-way ANOVA with Tukey's HSD *post hoc* test. Normality of ANOVA residuals was confirmed using the Shapiro–Wilk test for tensile index, porosity and surface roughness (*p* > 0.05). A minor normality violation (*p* < 0.05) was observed for air permeability, likely reflecting the left-censored 0 s values recorded for highly permeable samples (see air permeability); these samples were retained in the ANOVA. Parametric analysis was retained for all properties given the balanced experimental design and ANOVA's known robustness to minor violations of normality. To further investigate the independent and combined effects of non-wood fibre type, additive inclusion and substitution strategy on tensile index and porosity, a three-way factorial ANOVA was conducted on the nine non-wood blends (BAU and the single-fibre controls were excluded, as they do not fit the factorial design). The design comprised 18 formulations (3 fibre furnish × 2 additive × 3 substitution strategy combinations), each represented by three independent hand sheets, giving 54 observations analysed per response variable. Table S1 (SI) provides details of the complete design of the three-way ANOVA analysis.

### India as a case study

India was selected as the geographical region of interest for our study. As discussed, India represents a market with high usage of single use flexible plastics from FMCG companies. Bagasse, bamboo and old-corrugated cardboard were selected as non-wood fibres of interest, due to their relative high abundance across India, with approximately 45 Mt sugarcane bagasse,^27^ 4.6 Mt bamboo^28^ and 4.5 Mt of cardboard^26^ produced each year. These fibres are currently utilised as raw materials in the manufacturing of papers within India.^24^ In addition to non-wood fibres, India also utilises a range of additives in paper manufacturing, from starch^24^ to CNFs^29^,^30^ both of which are relevant to this study.

### Economic assessment

The production costs (USD per ton) of papers were calculated using the Fastmarkets RISI platform^24^ considering the cost values from April to June 2024. Fastmarkets RISI is the leading provider of pricing intelligence across the global pulp and paper markets, utilising primary data of process flows and unit costs, collected from market participants, including Indian paper mills. A market analysis specific to India was conducted to gather cost data for ‘Bleached Packaging’ and ‘Bleached Speciality’ papers within the ‘Speciality and Industry’ grades, to represent paper grades most similar to those considered as part of this study. The total cost of paper production from different mills was subdivided into the following categories: fibre, chemicals, furnish, energy, labor, delivery, materials and maintenance, capital expenditure (CAPEX) and selling, general and administrative costs (SG&A). Fibre, furnish and starch costs were obtained from average values based on the stated market analysis. Regional unit costs used to calculate paper production costs are described in Table 2. The cost of CNFs was obtained^31^ considering both energy and material costs and assuming production from bleached hardwood. CNF costs were averaged across the different production methodologies and adjusted for inflation using the chemical engineering plant cost index (CEPCI).

**Table 2:** Regional unit costs for calculating production costs of papers^a^

Parameter	Value
Depreciation method	Straight line
Cost year for analysis	2024
CEPCI 2011^b^	585.7
Bagasse ($US per Mt)	69.71
Bamboo ($US per Mt)	81.06
OCC ($US per Mt)	202.00
Northern bleached softwood kraft pulp ($US per Mt)	840.00
Hardwood, eucalyptus roundwood ($US per Mt)	149.21
Chemicals, packaging papermaking ($US per Mt)	7.06
Chemicals, other ($US per Mt)	2.94
Starch ($US per Mt)	423.24
CNF ($US per dry Mt)^c^	1542.50
Bagasse bleaching ($US per Mt)	28.89
Bagasse pulping ($US per Mt)	8.81
Bamboo bleaching ($US per Mt)	30.59
Bamboo pulping ($US per Mt)	9.32
Hardwood, eucalyptus, bleaching ($US per Mt)	33.99
Hardwood, eucalyptus, pulping ($US per Mt)	10.36
OCC pulping ($US per Mt)	2.49
Coal ($US per Mt)	108.64
Electricity ($US per kWh)	0.08
Oil No 6 ($US per BBL)	66.52
Biofuels ($US per Mt)	36.88
Hogfuel ($US per Mt)	52.68
Purchased steam ($US per K)	5.77
Labor, operators ($US per HR)	3.15
Labor, maintenance personnel ($US per HR)	3.28
Labor, exempt personnel ($US per HR)	6.30
Labor, non-exempt personnel ($US per HR)	3.60

a Data extracted from RISI Fastmarkets Platform,^24^ unless otherwise specified.

b CEPCI values sourced from ref. 32.

c CNF costs sourced from ref. 31.

Cost data were generated according to the following assumptions:

• Integrated mill for producing non-wood pulps and papers.

• Softwood pulp was imported and purchased as market pulp.

• Hardwood pulp was produced from hardwood roundwood.

• Finished papers were transported from integrated mills to Mumbai, India.

• The cost of chemicals, energy, labor, delivery, materials and maintenance, CAPEX and SG&A remains constant, regardless of feedstock selection.

• 1 Tonne of pulp generates 1 tonne of paper, with no losses.

The sensitivity of cost data was modelled using Monte Carlo simulation (*n* = 10 000), ensuring a relative error of the mean below 5% for a 95% confidence level.^33^ Each cost parameter was sampled independently from a normal distribution; no correlations between input parameters were modelled. The mean and standard deviation used to define the normal distribution for each sampled cost parameter are reported in Table 7, allowing the Monte Carlo simulation inputs to be reconstructed. The sensitivity of chemicals, energy, labor, delivery, materials and maintenance, CAPEX and SG&A costs was assessed across 16 different paper machines, across 7 paper mills in India. This captures price variability in chemical, energy, labor, delivery, maintenance, CAPEX and SG&A costs but not the structural assumption that these costs remain constant regardless of feedstock selection, which is acknowledged as a separate limitation. The sensitivity of fibre, furnish and starch costs was assessed over time; every quarter between Q1 2014 and Q3 2024. The sensitivity of CNF costs was assessed across the different CNF production methods presented by Spence *et al.*^31^

### Environmental impact assessment

The environmental impact assessment of paper production was calculated using an attributional LCA according to the EN ISO 14040 (2006)^34^ LCA framework: (i) study goal and scope, (ii) life cycle inventory, (iii) life cycle impact assessment and (iv) interpretation. The proposed cradle-to-gate system boundary of the study is outlined in Fig. 2, highlighting the goal and scope of the study. India is assumed as the proposed geographical area, with all hardwood, bagasse, bamboo, recycled fibres and starch assumed locally sourced. Given the uncertainties in the CNF supply chain, CNF production was instead assumed to follow a global supply chain, with a global electricity mix applied within the LCA model. Softwood market pulp is assumed to be sourced from Canada^35^ in the form of softwood kraft pulp^24^ and is transported by sea ferry across a distance of 25 679 km, calculated using typical sea freight routes.^36^

**Fig. 2:**
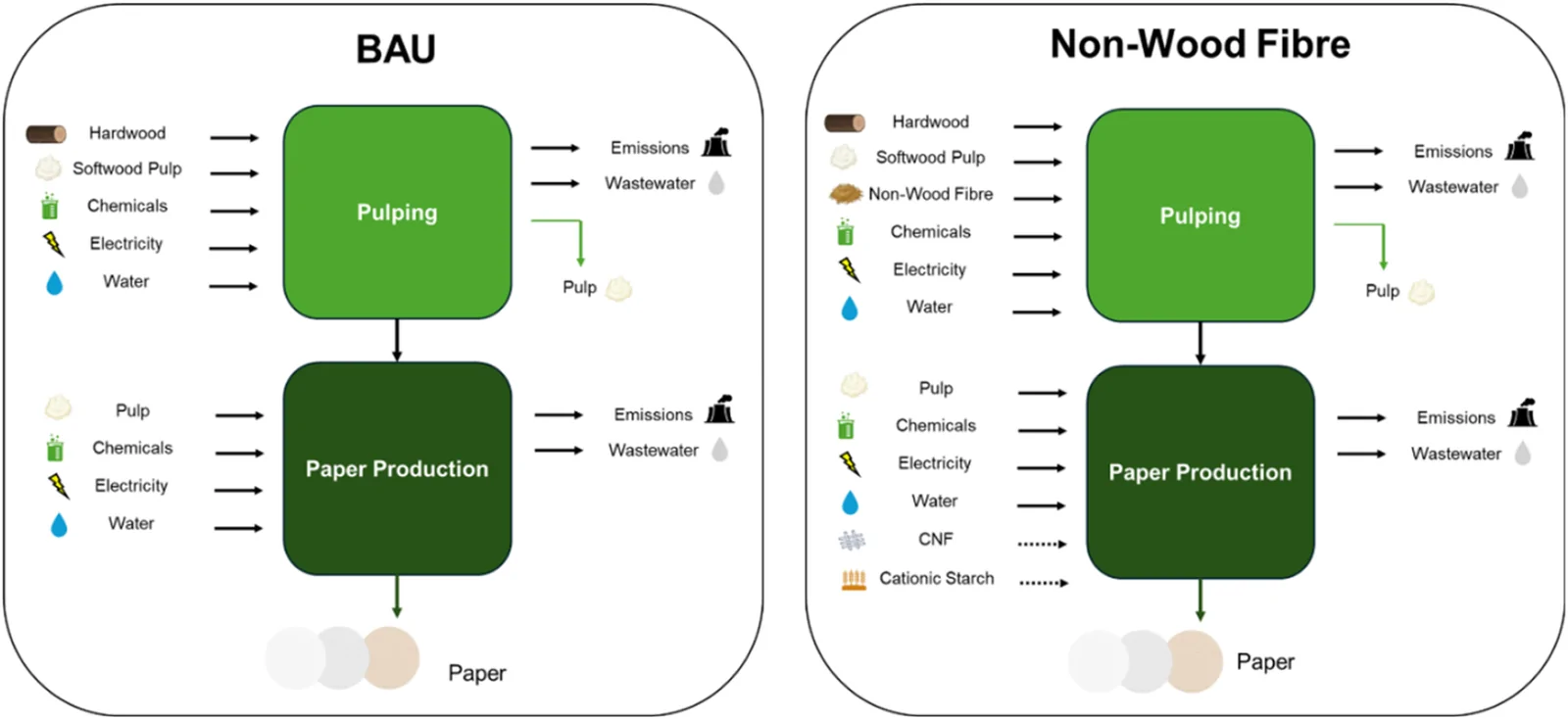
Proposed system boundary for non-wood fibre containing papers, compared to business-as-usual papers.

The functional unit of the process is one tonne of dry, uncoated paper produced. For additive-containing papers, inventories were normalised on a mass basis to 1 kg of dry paper (0.965 kg fibre, 0.025 kg CNF and 0.010 kg cationic starch). Additive retention was assumed to be 100%. As this study adopts a cradle-to-gate scope, no specific packaging format was assumed. Different formats require different functional properties and coating amounts, so a mass-based functional unit was selected to enable comparison across formulations regardless of packaging format. The life cycle inventories used for the life cycle impact assessments are described in Tables 3 and 4, with a detailed breakdown of each inventory and associated Ecoinvent inventories described in Tables S2–S8 (SI). Ecoinvent inventory ‘kraft paper {RER}|kraft paper production|cut-off, *U*’ was used as a baseline inventory, but modified to reflect an Indian geography and energy mix.^24^ Bagasse and bamboo fibre inventories were derived from literature sources (Tables S2 and S4, respectively; SI). Corresponding pulp production inventories were derived using the FisherSolve platform^37^ (Tables S3 and S5, respectively; SI), implying pulp yields of 30.9% (bagasse) and 41.0% (bamboo). These yields differ from the market-average yields reported in Table 6 (43% bagasse and 45% bamboo; sourced from RISI Fastmarkets^24^), which are used specifically for feedstock cost calculations, not the LCA mass balance. OCC inventory data were derived from ref. 38. Overall inventories were deduced through mass balance between different fibre mixes. It is assumed that 1 kg of pulp produces 1 kg paper, with no losses. The density of hardwood was assumed as 1 *m*^3^ = 740 kg.^39^ An integrated mill is assumed (producing both pulp and paper on-site), with the energy distribution assumed as 60% towards pulp production and 40% towards paper production.^40^ India's energy mix is largely driven by fossil fuels, with approximately 90% of India's energy consumption attributed to coal, gas and oil in 2024.^41^ India is most dependent on coal as the fuel source, which represented approximately 57% of India's total commercial primary energy consumption.^41^

**Table 3:** Life cycle inventory normalised to 1 kg paper produced, comparing business-as-usual (BAU) and non-wood fibre containing papers without additives

Input/output	BAU	BAG-SWHW	BAG-SW	BAG-HW	BAM-SWHW	BAM-SW	BAM-HW	OCC-SWHW	OCC-SW	OCC-HW
**Raw materials**										
Bagasse/kg	0	0.647	0.647	0.647	0	0	0	0	0	0
Bamboo/kg	0	0	0	0	0.488	0.488	0.488	0	0	0
OCC/kg^a^	0	0	0	0	0	0	0	0	0	0
Softwood pulp/kg	0.300	0.240	0.100	0.300	0.240	0.100	0.300	0.240	0.100	0.300
Hardwood, roundwood/m^3^	0.00205	0.00164	0.00205	0.00146	0.00164	0.00205	0.00146	0.00164	0.00205	0.00146
Chemicals/kg	0.0860	0.0964	0.0964	0.0964	0.0998	0.0998	0.0998	0.0716	0.0716	0.0716

**Utilities**										
Heat/MJ	9.25	8.14	8.14	8.14	8.14	8.14	8.14	8.14	8.14	8.14
Electricity/kWh	0	0	0	0	0.0464	0.0464	0.0464	0.0148	0.0148	0.0148
Oil/kg	0	0.00226	0.00226	0.00226	0.00252	0.00252	0.00252	8.26 × 10^−5^	8.26 × 10^−5^	8.26 × 10^−5^
Coal/kg	0	0.0142	0.0142	0.0142	0.001	0.001	0.001	5.38 × 10^−5^	5.38 × 10^−5^	5.38 × 10^−5^
Natural gas/m^3^	0	0.00307	0.00307	0.00307	0.00265	0.00265	0.00265	0.00254	0.00254	0.00254
Biogas/m^3^	0	0.00155	0.00155	0.00155	0	0	0	0	0	0
Water/m^3^	0.06	0.06	0.06	0.06	0.06	0.06	0.06	0.06	0.06	0.06

**Transport**										
Sea ferry/tkm	7.7	6.2	2.6	7.7	6.2	2.6	7.7	6.2	2.6	7.7

**Emissions**										
CO_2_ emissions/kg	0.13	0.17	0.17	0.17	0.13	0.13	0.13	0.12	0.12	0.12

**Products**										
Paper/kg	1.00	1.00	1.00	1.00	1.00	1.00	1.00	1.00	1.00	1.00

a Assumed burden-free.

**Table 4:** Life cycle inventory normalised to 1 kg paper produced comparing non-wood fibre containing papers with additives

Input/output	BAG-SWHW	BAG-SW	BAG-HW	BAM-SWHW	BAM-SW	BAM-HW	OCC-SWHW	OCC-SW	OCC-HW
**Raw materials**									
Bagasse/kg	0.625	0.625	0.625	0	0	0	0	0	0
Bamboo/kg	0	0	0	0.471	0.471	0.471	0	0	0
OCC/kg^a^	0	0	0	0	0	0	0	0	0
Softwood pulp/kg	0.232	0.0965	0.290	0.232	0.0965	0.290	0.232	0.0965	0.290
Hardwood, roundwood/m^3^	0.00159	0.00198	0.00142	0.00159	0.00198	0.00142	0.00159	0.00198	0.00142
Chemicals/kg	0.0964	0.0964	0.0964	0.0998	0.0998	0.0998	0.0716	0.0716	0.0716
CNF/kg	0.025	0.025	0.025	0.025	0.025	0.025	0.025	0.025	0.025
Starch/kg	0.01	0.01	0.01	0.01	0.01	0.01	0.01	0.01	0.01

**Utilities**									
Heat/MJ	8.14	8.14	8.14	8.14	8.14	8.14	8.14	8.14	8.14
Electricity/kWh	0	0	0	0.0464	0.0464	0.0464	0.0148	0.0148	0.0148
Oil/kg	0.00226	0.00226	0.00226	0.00252	0.00252	0.00252	8.26 × 10^−5^	8.26 × 10^−5^	8.26 × 10^−5^
Coal/kg	0.0142	0.0142	0.0142	0.001	0.001	0.001	5.38 × 10^−5^	5.38 × 10^−5^	5.38 × 10^−5^
Natural gas/m^3^	0.00307	0.00307	0.00307	0.00265	0.00265	0.00265	0.00254	0.00254	0.00254
Biogas/m^3^	0.00155	0.00155	0.00155	0	0	0	0	0	0
Water/m^3^	0.06	0.06	0.06	0.06	0.06	0.06	0.06	0.06	0.06

**Transport**									
Sea ferry/tkm	5.98	2.51	7.43	5.98	2.51	7.43	5.98	2.51	7.43

**Emissions**									
CO_2_ emissions/kg	0.17	0.17	0.17	0.13	0.13	0.13	0.12	0.12	0.12

**Products**									
Paper/kg	1.00	1.00	1.00	1.00	1.00	1.00	1.00	1.00	1.00

a Assumed burden-free.

The environmental impact was characterised using the ReCiPe 2016 H (hierarchist) model at the midpoint stage, calculated using SimaPro V8. The midpoint characterisation model assesses 18 impact categories: global warming, ozone depletion, ionizing radiation, ozone formation (human health), particulate matter formation, ozone formation (terrestrial ecosystems), acidification, freshwater eutrophication, marine eutrophication, terrestrial ecotoxicity, freshwater ecotoxicity, marine ecotoxicity, human carcinogenic toxicity, human non-carcinogenic toxicity, land utilization, resource scarcity, fossil scarcity and water consumption.

## Results and discussion

### Pulp characteristics

Pulp characteristics are described in Table 5. The compositions of main fibre components (cellulose, hemicellulose and lignin) of softwood and hardwood pulps are typically similar. However, the length of softwood fibres (2.24 mm) were higher, compared to that of hardwood fibres (0.81 mm), reflecting similar conclusions presented by Antonsson *et al.*^42^ Softwood fibre lengths are described as around three times the length of hardwood fibres.^43^ Tracheid cells, which contain the majority of softwood fibres, are typically longer than hardwood fibres, as they provide dual functionality, contributing towards bulk properties and water conduction, whereas hardwood fibres only minimally contribute to water conduction through the tree.^44^ Non-wood pulps have been previously described to contain a comparatively higher hemicellulose content and lower lignin content compared to wood-based pulps.^18,45^ However, Table 5 shows that the lignin contents of bagasse (12.4%) and bamboo (10.9%) were higher than those of softwood (7.2%) or hardwood (4.2%). Softwood and hardwood pulps were purchased as bleached kraft pulps, whereas the custom pulping of bagasse and bamboo did not involve a bleaching stage. Bleaching is a process used to remove lignin from pulps,^40^ hence the increased lignin content of the unbleached non-wood pulps. Table 5 shows that bamboo displayed a longer fibre length (1.40 mm) compared to bagasse (1.06 mm), reflecting conclusions of previous studies.^18,46^ The fibre length of OCC is comparable to that of bagasse. However, the (holo)cellulose content was lower in OCC than all other pulps considered, likely due to the presence of high-ash fillers or additives. Table 5 shows an increased content of fines for OCC compared to other pulps. During the recycling process, fibres are subject to mechanical disintegration, refining and high temperatures, which can effectively degrade fibres,^47^ resulting in increased prevalence of fines.^48^

Composition of main fibre components and fibre morphology of pulps

**Table:** 

Biochemical analysis	Softwood	Hardwood	Bagasse	Bamboo	OCC
Cellulose, wt%	76.4 ± 0.2	76.2 ± 0.5	71.4 ± 0.1	71.8 ± 0.3	53.1 ± 0.2
Hemicellulose, wt%	15.2 ± 0.2	16.3 ± 0.2	21.6 ± 0.1	20.4 ± 0.2	12.7 ± 0.1
Lignin, wt%	7.2 ± 0.2	4.2 ± 0.4	12.4 ± 0.4	10.9 ± 0.1	13.5 ± 0.1

**Table:** 

Fibre morphology	Softwood	Hardwood	Bagasse	Bamboo	OCC
Length, mm	2.24 ± 0.02	0.81 ± 0.00	1.06 ± 0.03	1.40 ± 0.01	1.10 ± 0.01
Width, µm	28.1 ± 0.1	18.8 ± 0.1	22.6 ± 0.0	17.1 ± 0.0	27.6 ± 0.1
Length : width ratio	80	43	47	82	40
Fines, %	16.2 ± 1.9	14.6 ± 0.5	8.6 ± 0.7	10.0 ± 0.0	48.6 ± 0.5

### Physical properties of hand sheets

Fig. 3 shows the physical characteristics of the hand sheets. These properties were selected to gain an understanding of how combinations of fibre blends and additives influence the structural integrity of the papers.

**Fig. 3:**
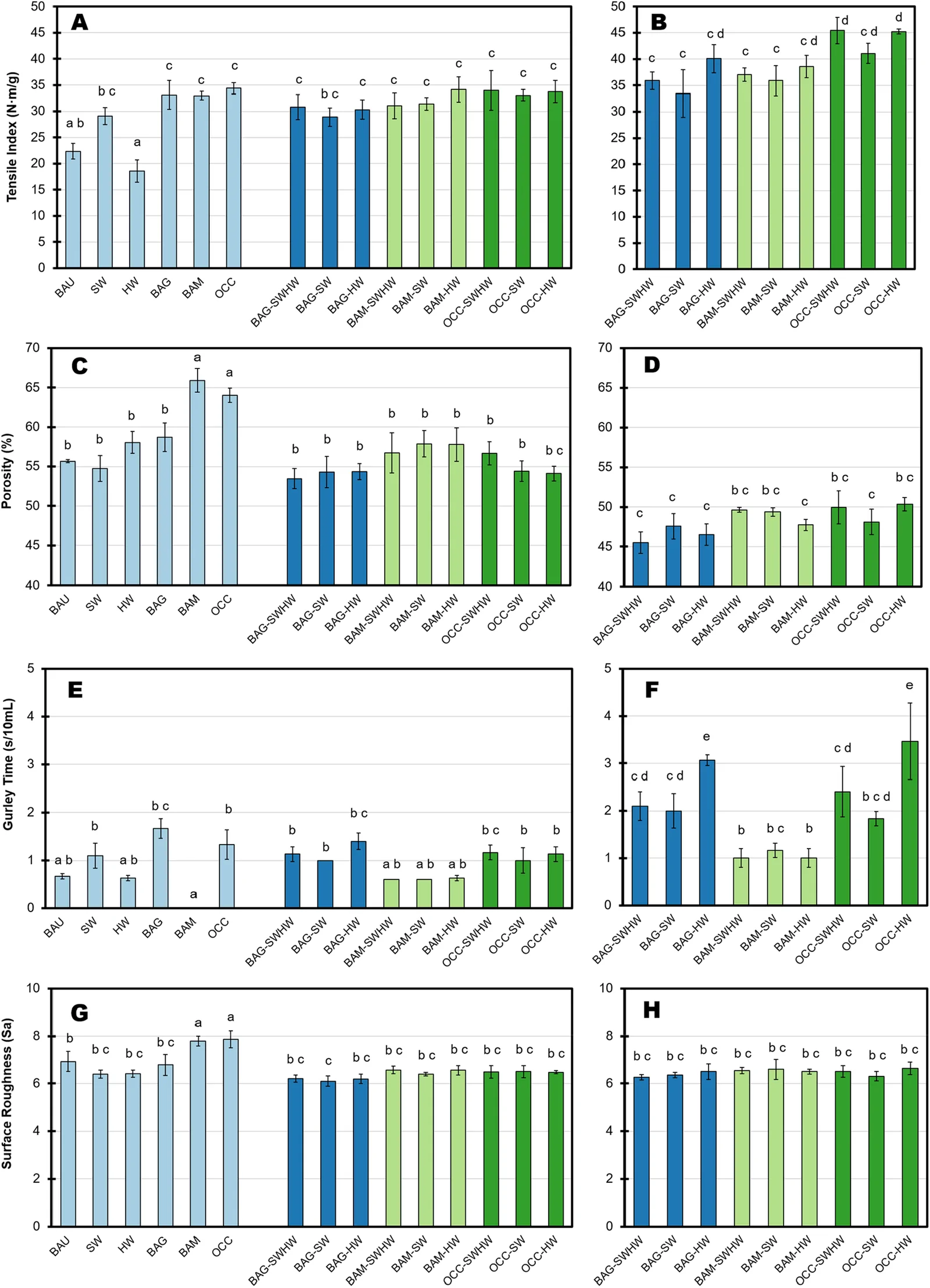
Physical properties of hand sheets: tensile index without additives (A), tensile index with additives (B), porosity without additives (C), porosity with additives (D), Gurley time without additives (E), Gurley time with additives (F), surface roughness without additives (G) and surface roughness with additives (H). Different letters above bars indicate statistically significant differences between formulations (Tukey’s HSD *post hoc* test *p* < 0.05); shared letters indicate no significant difference.

One-way ANOVA analysis highlights significant (*p* < 0.001) differences in tensile index across the 24 formulations. The tensile index of BAU paper was 22.4 N m g^−1^; logically between that of SW (29.1 N m g^−1^) and HW (18.6 N m g^−1^). SW pulps typically have a higher tensile index than HW pulps.^42^ Longer SW fibres offer a greater surface area for inter-fibre physical crosslinking,^43^ resulting in increased strength. Table 5 shows that the SW used in this study has a longer fibre length than HW fibres. Fig. 3A shows that the tensile index values of BAG (33.1 N m g^−1^), BAM (33.0 N m g^−1^) and OCC (34.4 N m g^−1^) are all significantly (*p* < 0.05) higher than that of BAU. BAG displays a similar (*p* > 0.05) tensile index to SW (Fig. 3A), despite a reduced fibre length (Table 5), indicating that additional physical characteristics of fibres can contribute towards mechanical properties of hand sheets.

The degree of fibrillation also affects the physical properties of pulps. Higher fibrillation of pulps increases the surface area for inter-fibre hydrogen bonding,^49,50^ impacting the physical properties, such as an increased tensile index. Mechanical treatments, such as beating or refining, are utilised within the paper industry to improve fibrillation, which can be represented as a measurement of freeness, drainage rate of a pulp suspension.^49^ The pulps considered as part of this study were unrefined and had not been subjected to any prior mechanical treatment. Banavath *et al.*^49^ found that the tensile index of unrefined bagasse pulp (45.9 N m g^−1^) was higher than that of unrefined softwood pulp (10.5 N m g^−1^), linked to a higher water retention capacity (in this case, lower CSF freeness) of the bagasse pulp, indicating a higher degree of fibrillation and inter-fibre bonding. This supports the results of Fig. 3A and suggests that unrefined bagasse pulp may have a higher degree of fibrillation compared to unrefined SW pulp, although fibrillation was not directly measured in this study. OCC displays similar (*p* > 0.05) tensile index to SW, despite a shorter fibre length. Table 5 indicates that the concentration of fines in OCC pulp (48.6%) is significantly higher than that in the other pulps considered (8.6–16.2%). Higher concentrations of fines result in an increased tensile index by facilitating inter-fibre bonding.^51^ Although, no significant improvements in tensile index were observed beyond 30% fines concentration.^51^ Similarly, Ferdous *et al.*^46^ reported that pulp fines had a positive influence on the tensile index across a range of unrefined non-wood pulps. However, the same trend was not observed for refined pulps.

All hand sheets containing 20% inclusion of non-wood fibre displayed a significantly (*p* < 0.05) higher tensile index compared to BAU, with the exception of BAG-SW. Zhang *et al.*^52^ reported a positive correlation between the hemicellulose content of fibres and tensile strength of paper, as hemicellulose acts as a binding agent between fibres. The hemicellulose contents of bagasse (21.6%) and bamboo pulps (20.4%) were markedly higher than that of SW (15.2%) and HW pulps (16.3%), evidenced in Table 5, which could act as a contributory factor to the increased tensile index of hand sheets containing blends of non-wood fibres.

Three-way factorial ANOVA identified additive inclusion and non-wood fibre type as significant (*p* < 0.001) drivers of tensile index, whereas no significant (*p* > 0.05) differences in tensile index were reported regardless of whether SW, HW or SWHW fraction of virgin wood fibre is substituted with non-wood fibre. Fig. 3B shows that inclusion of additives increased tensile index by 16–32% (BAG), 13–19% (BAM) and 24–34% (OCC), compared to equivalent hand sheets without additives, with OCC-containing hand sheets demonstrating significantly (*p* < 0.001) greater improvements than BAG or BAM-containing samples. In a similar mechanism to mechanical refining, the inclusion of CNFs is thought to increase the surface area for inter-fibre hydrogen bonding, resulting in an increase in the strength of papers.^53^ It should be noted that the degree of fibrillation, water retention value, and drainage behaviour were not directly measured as part of this study; their contributions to the trends discussed above are therefore inferred, not confirmed experimentally.

Fig. 3C and D show the porosity, or voidage, of the hand sheets. Less porous papers are more desirable for packaging applications, providing a compatible surface for applying barrier coatings to protect products from environmental factors, such as moisture ingress. One-way ANOVA analysis highlights significant (*p* < 0.001) differences in porosity across the 24 formulations. The porosity of the BAU hand sheet was 55.7%, significantly (*p* < 0.05) lower than that of BAM and OCC. However, hand sheet blends containing 20% non-wood fibre without additives, all displayed a similar porosity compared to BAU, likely a result of only substituting a small portion (20%) of virgin wood fibres. Fig. 3D shows that inclusion of additives decreased the porosity of BAG, BAM and OCC blends by 14–17%, 14–21% and 7–13%, respectively. Reductions in porosity are likely due to CNFs, which facilitate inter-fibre bonding^54^ and pore filling. Similarly to tensile index, there was no significant difference in the porosity of non-wood blends, regardless of whether SW, HW or SWHW is substituted.

Minimising air permeability of papers is also desirable to enhance compatibility of applying barrier coatings and is often related to the porosity of papers. Fig. 3E and F shows the Gurley time of hand sheets, where a higher Gurley time corresponds to a lower air permeability. One-way ANOVA analysis highlights significant (*p* < 0.001) differences in air permeability across the 24 formulations. BAG (1.7 s/10 mL) and OCC (1.3 s/10 mL) display lower air permeability compared to BAU (0.7 s/10 mL), whereas BAM exhibited such high air permeability that it could not be measured using the Gurley instrument. Compared to BAU, BAG and OCC blends containing additives show a significantly (*p* < 0.05) reduced air permeability. However, BAM-containing hand sheets show a similar air permeability to BAU, though this comparison may underestimate the true difference given the left-censored (0 s) nature of many BAM values.

Fig. 3G and H displays the surface roughness of hand sheets. One-way ANOVA analysis highlights significant (*p* < 0.001) differences in surface roughness across the 24 formulations. Although BAM and OCC display the highest surface roughness, this is minimised when utilised as blends. All non-wood fibre blends show no significant (*p* > 0.05) difference in surface roughness compared to BAU in the presence of additives. Fig. S2 (SI) provides images from confocal microscopy analysis, which show that individual fibres in BAM and OCC hand sheets are more pronounced than within BAU, resulting in a rougher surface. Surface roughness is not significantly (*p* > 0.05) affected regardless of SW, HW or SWHW substitution with non-wood fibre.

Overall, the results of Fig. 3 suggest that, from a physical characteristics perspective, properties of hand sheets improve or remain similar to those of BAU regardless of SW, HW or SWHW substitution with 20% non-wood fibre inclusion. Blending with BAG or OCC may be more favourable than BAM, due to the higher air permeability observed with bamboo fibres, noting that comparisons involving BAM remain subject to the left-censoring limitation described above. In this study, laboratory-scale hand sheets were used to infer the fundamental effects of pulp blends and additive inclusion on paper properties. During industrial-scale production, additional processing steps further modify these properties, including machine-direction fibre orientation, industrial refining, retention systems, wet pressing, cylinder drying and calendering. As such, the laboratory values reported here should not be treated as direct predictions of industrial-scale paper performance.

### Cost analysis

The total production costs (USD per ton paper) and relative inventory contributions of both BAU and non-wood fibre blends are shown in Fig. 4. The production cost of the BAU paper was $868 USD per ton, with the major contributors to the cost being fibre (51%), energy (11%) and SG&A (9%) costs. Fig. 4A shows that the most significant reduction in costs was observed during SW substitution with non-wood fibres. Substitution of SW with non-wood fibre results in a 22–24% reduction in fibre cost; driving an 11–12% reduction in the total cost of the paper to $761–$770 USD per ton. Cost savings were similar regardless of which non-wood fibre was utilised (BAG, BAM, or OCC). Alternatively, no significant cost saving was observed when substituting HW with non-wood fibres, resulting in a 1–2% reduction in cost compared to BAU. Table 6 highlights the higher costs producing papers using softwood market pulp compared to hardwood, bagasse, bamboo or OCC biomass. Fig. 4B shows the production costs of papers, including the costs of additives.

**Fig. 4:**
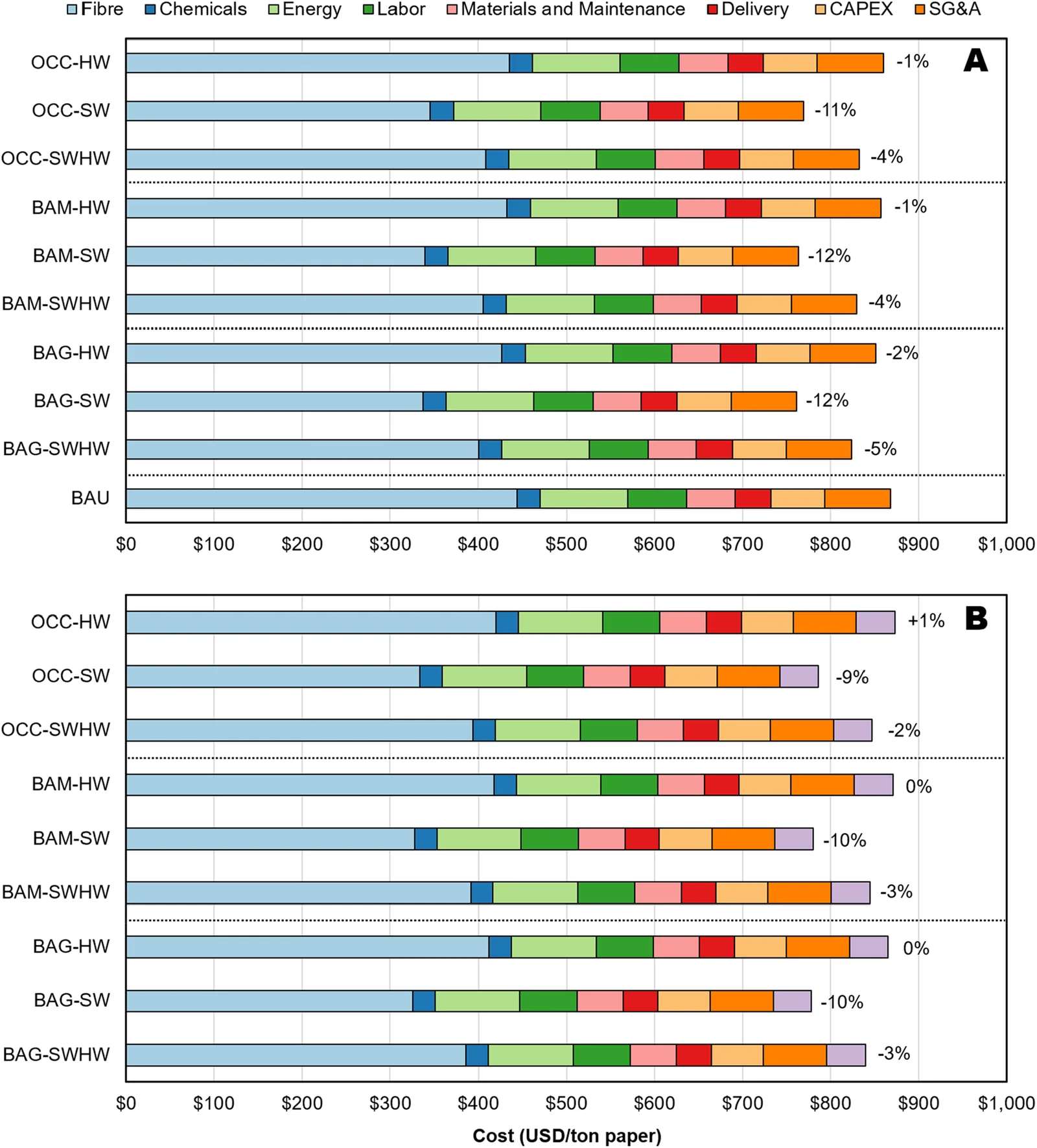
Total production cost of papers without additives (A) and including additives (B). Percentage values represent the relative change in production cost compared to BAU.

**Table 6:** Total cost for each fibre type, based on the quantity of biomass required to produce one ton of mono-material paper

Feedstock	Unit cost (USD per ton)	Pulp yield from feedstock (%)^c^	Feedstock mass (ton per ton paper)	Fibre cost (USD per ton paper)
Softwood market pulp^a^	759	—	1.00	759
Hardwood^a^	121	46%	2.17	262
Bagasse^a^	78	43%	2.33	182
Bamboo^a^	98	45%	2.22	218
OCC^b^	207	79%	1.27	262

a Bleached.

b Unbleached.

c Data extracted from RISI Fastmarkets; yields used for feedstock cost calculation only. OCC = old corrugated cardboard.

Additive costs total $44 USD per ton paper, of which 89% of this cost is associated with CNFs. The unit cost of CNFs ($1543 USD per ton) is significantly higher than that of starch ($492 USD per ton) driven by the large energy demand of CNF production. CNF energy demand is typically between 1150 and 8755 kWh per ton, depending on production method,^31^ whereas the energy demand of starch production is approximately 702 kWh per ton.^55^ Within Asia, around 70–85% of starch production cost is a result of raw material costs.^56^ Substituting SW with non-wood fibres, with the inclusion of additives results in a 9–10% cost saving compared to BAU, whereas substitution of HW with non-wood fibres in the presence of additives results in cost parity compared to BAU. Overall, SW substitution represents the greatest cost saving when integrating non-wood fibres into papers. However, Fig. 5 shows large variations in the total production costs of papers. Although the median cost values for SW substitution are lower than BAU or HW substitution, it is difficult to draw definitive conclusions with such high variability of costs.

**Fig. 5:**
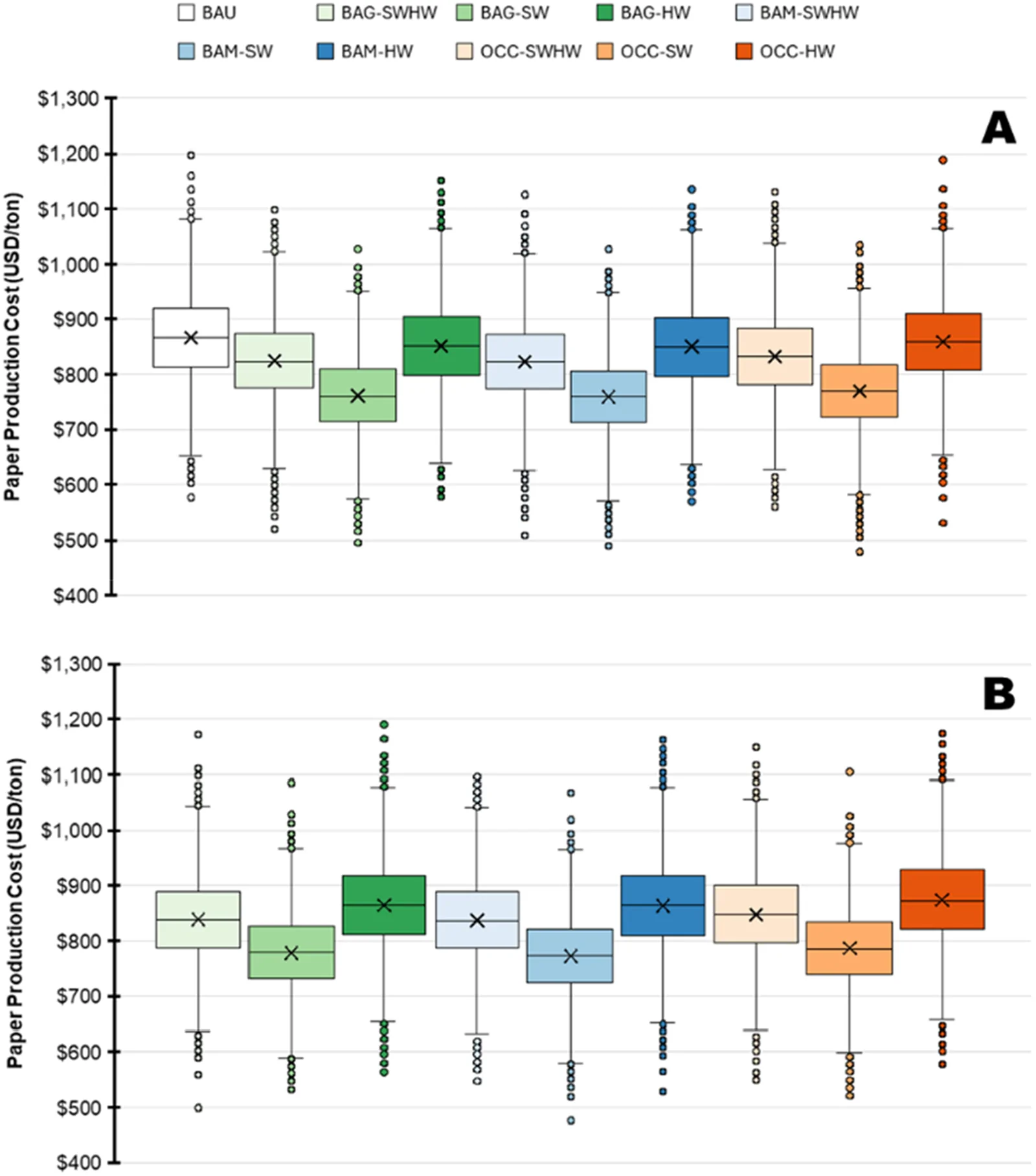
Sensitivity analysis of production costs of papers without additives (A) and with additives (B) using Monte Carlo simulation (*n* = 10 000).

Fig. 6 and 7 describe the proportional contribution of each category to the total cost sensitivity of paper, without and with additives respectively. Fig. 6 shows that the greatest contributors to the variability of BAU costs are SG&A (32%), fibre (29%) and energy (17%), with similar hotspots identified for HW substitution with BAG, BAM or OCC. Variations in SG&A and energy costs are associated with differences in operational conditions and geographical location and the sources of energy mixes across the in-scope mills analysed for this study, whereas, variability in fibre costs is attributed to temporal fluctuations in Indian market costs;^24^ for example, during Q3 2019, the unit cost of bleached softwood market pulp was $585 USD per ton, compared to $1040 USD per ton during Q2 2022. Substituting SW with non-wood fibres reduced the proportional sensitivity contribution of fibres from 29% to 10–11%; indicating that substituting SW with non-wood fibres could result in both cost reduction and a less variable cost. Fig. 7 includes the proportional contribution of additives to the variability of paper production costs. Despite high variability in CNF costs (Table 7), Fig. 7 suggests that additives only contribute towards 5–6% of the overall paper cost variability, at the specified inclusion levels.

**Fig. 6:**
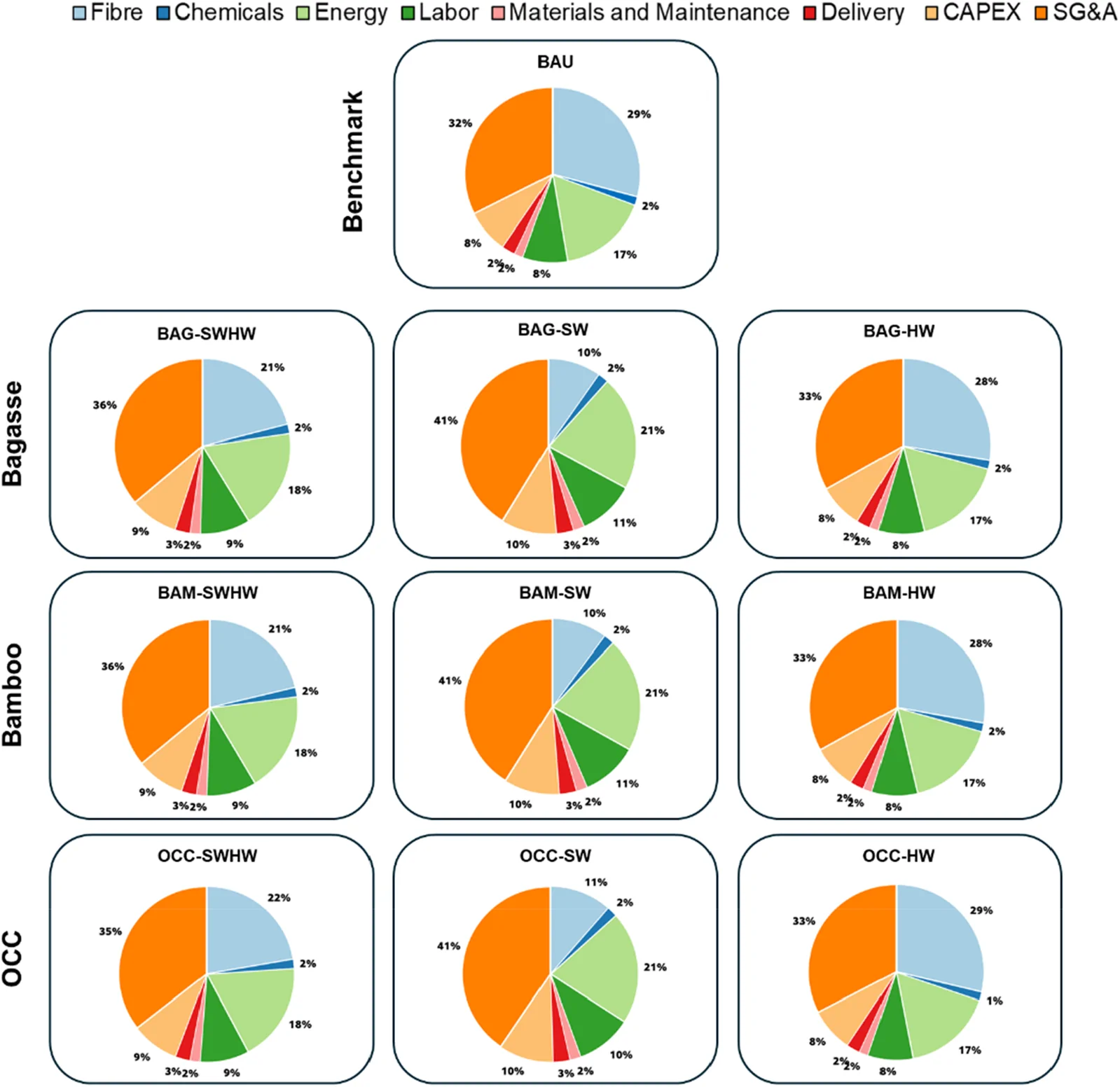
Proportional impacts of total cost sensitivity for papers without additives.

**Fig. 7:**
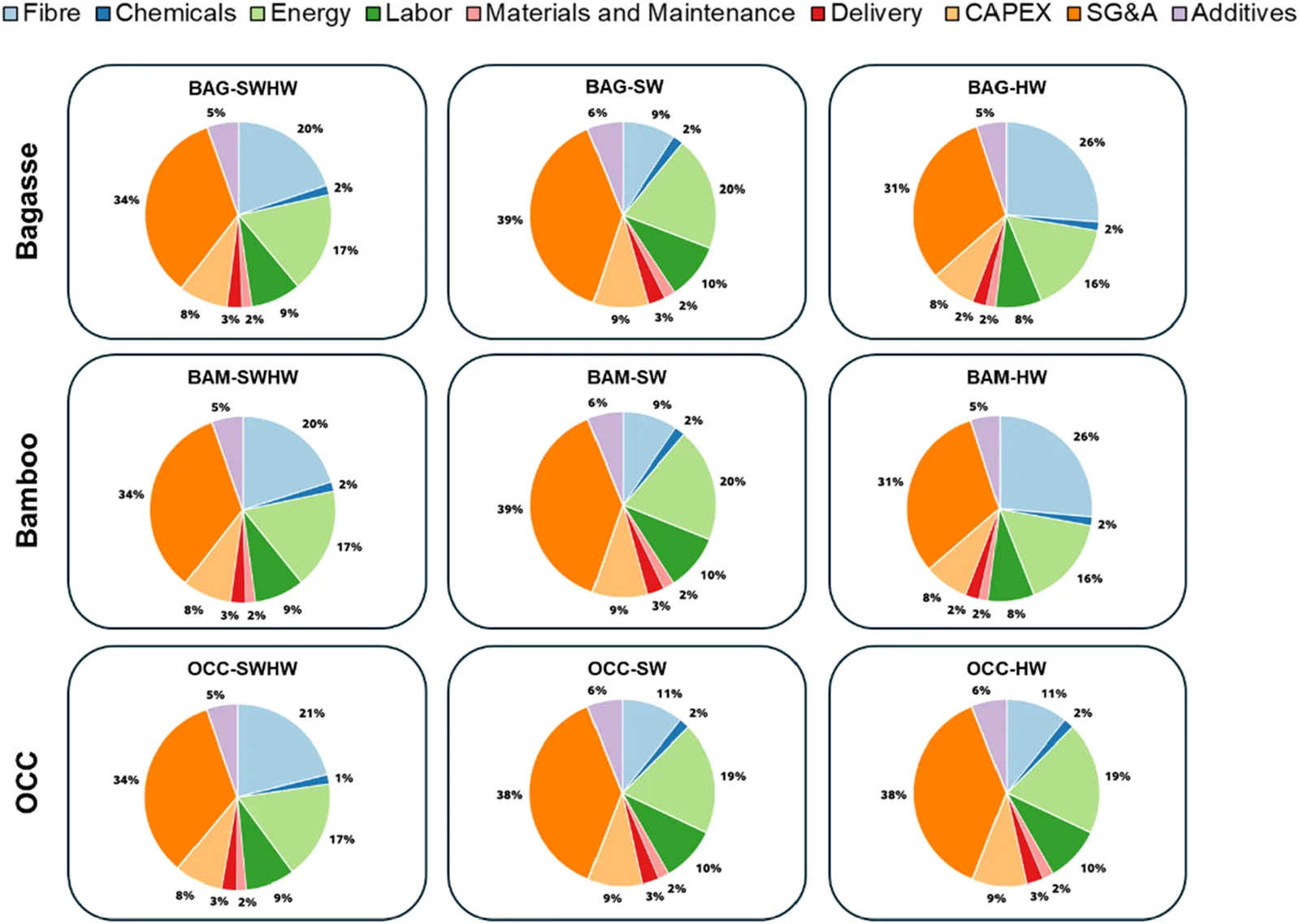
Proportional impacts of total cost sensitivity for papers with additives.

Average, minimum and maximum costs, with standard deviation, used to define the Monte Carlo simulation

**Table:** 

Item	Cost (USD per ton paper)			
	Minimum	Maximum	Average	Standard deviation
**Fibre**				
Bagasse	161.87	219.02	182.14	16.95
Bamboo	179.95	281.05	217.15	28.05
OCC	147.32	381.00	262.43	51.34
Hardwood (roundwood)	227.48	326.63	262.15	24.61
Softwood market pulp	585.00	1040.00	758.49	128.84

**Furnish**				
Hardwood bleaching	32.22	41.97	37.65	2.20
Hardwood pulping	8.02	11.39	9.20	0.72
Bagasse bleaching	27.39	39.24	33.49	2.98
Bagasse pulping	7.54	12.80	9.36	1.76
Bamboo bleaching	21.55	32.23	27.14	3.40
Bamboo pulping	5.42	10.25	7.78	1.47
OCC pulping	2.43	4.08	3.07	0.50

**OPEX**				
Chemicals	10.04	31.90	26.43	9.77
Energy	55.32	159.64	99.67	32.42
Labor	32.20	94.67	67.24	22.88
Materials and maintenance	35.08	64.91	54.81	9.96
Delivery	19.57	51.65	40.53	12.47
CAPEX	37.85	119.74	61.48	22.25
SG&A	30.00	166.49	74.16	45.19

**Table:** 

	Cost (USD per ton)			
	Minimum	Maximum	Average	Standard deviation
CNF	1016.06	2673.12	1542.50	700.26
Starch	425.00	534.45	492.04	29.67

### Life cycle assessment

The environmental impacts of producing non-wood fibre-containing papers without additives are presented in Fig. 8A and S3 (SI). Overall, the main hotspots identified were associated with the energy consumption of paper production and the transportation of softwood pulp to India. Energy consumption accounted for the majority of impacts across water pollution impact categories, contributing 67–79% of freshwater eutrophication, freshwater ecotoxicity and marine ecotoxicity impacts and 48–56% of marine eutrophication impacts. Human health impacts (both carcinogenic and non-carcinogenic) were also largely attributed (52–87%) to energy consumption. Coal is the dominant fuel source within India, representing around 46% of total energy supply,^57^ according to 2023 figures. This value represents a lower share than reported elsewhere in the manuscript, reflecting IEA's inclusion of biomass within India's total energy supply. Coal is associated with the leaching of hazardous metal ions from gangue; a solid waste by-product generated during the mining process.^58^ During the combustion of coal, hazardous species are contained in the fly ash, which can leach into water-ways resulting in water pollution and human health environmental impacts.^59^ Alternatively, Fig. S3 (SI) shows that the greatest contribution towards terrestrial acidification (57–79%), fine particulate formation (46–71%), ozone formation (human health) (56–78%) and ozone formation (terrestrial ecosystems) (56–79%) impacts were a result of transportation, largely imports of softwood pulp to India. Ocean freight releases nitrogen oxides, sulphur oxides, carbon dioxide, carbon monoxide, volatile organic compounds and particulate matter, which contribute to the aforementioned environmental impacts.^60^

**Fig. 8:**
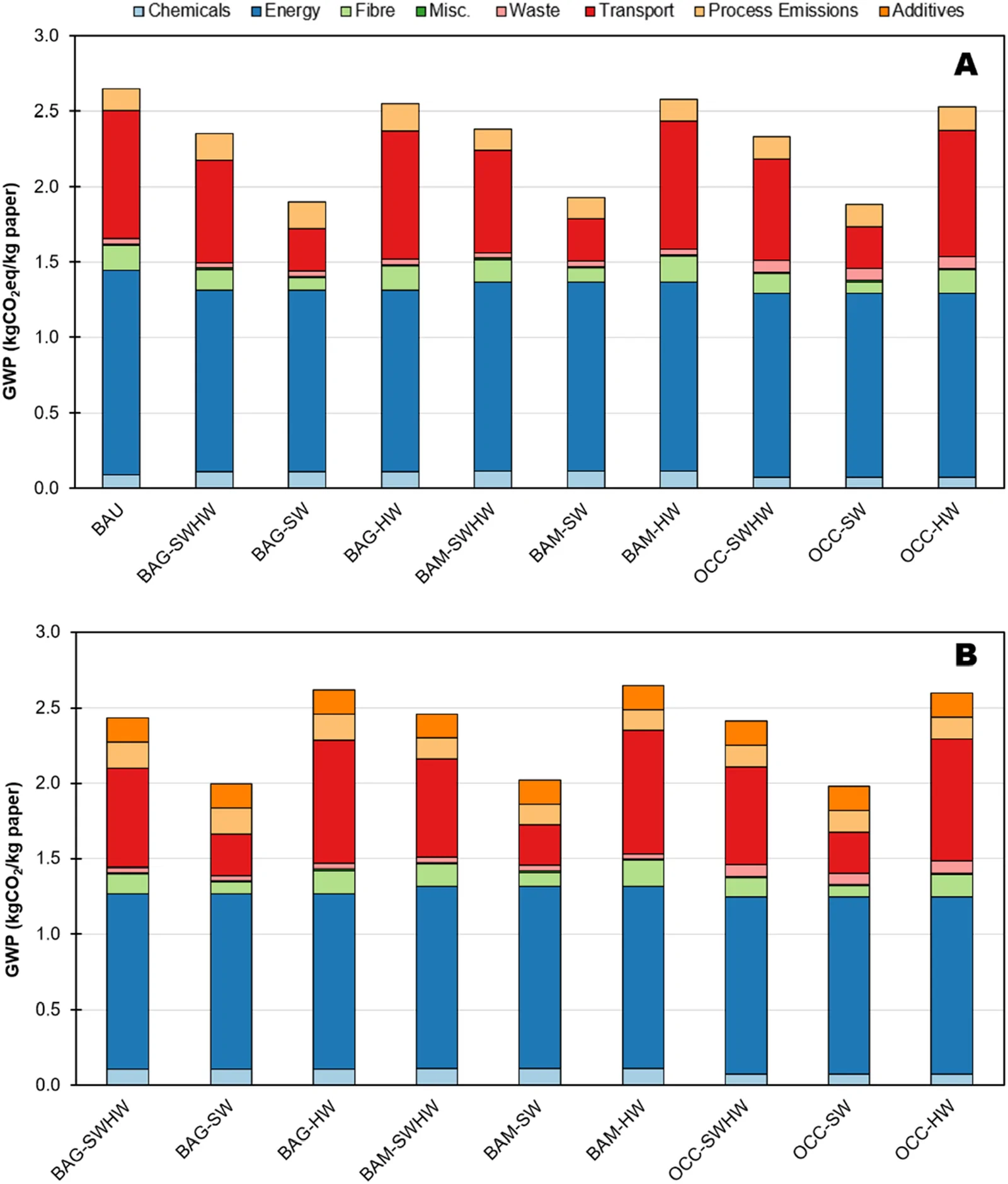
Global warming potential (GWP) of papers without additives (A) and with additives (B).

Fig. 8A shows that the global warming potential (GWP) of the BAU scenario was 2.65 kg CO_2_ eq. per kg paper, largely due to contributions from energy (51%) and transport (32%) impacts. Energy-related environmental impacts are largely due to India's dependency on coal as a primary energy source, whereas transport impacts are driven by CO_2_ emissions from fossil-derived fuels for freight vessels.^61^ These values represent the impact of the uncoated paper substrate; additional processing required for final packaging functionality, such as barrier coatings, falls outside the study's cradle-to-gate scope. Replacing SW with non-wood fibres provides the greatest reduction in GWP compared to BAU (Fig. 8A). A 27–29% reduction in GWP was observed when SW was replaced with bagasse (1.90 kg CO_2_ eq. per kg), bamboo (1.93 kg CO_2_ eq. per kg) or OCC (1.88 kg CO_2_ eq. per kg). Reductions in GWP were primarily a result of a 67% reduction in transport impacts, due to reduced dependence on SW pulp imports. Substituting HW with bagasse, bamboo or OCC results in a 3–5% reduction in GWP impact compared to the BAU scenario. Therefore, replacing SW with non-wood fibres is a more effective strategy to reduce GWP, compared to HW substitution, linked to decarbonisation through utilising localised supply chains. These environmental impacts reflect a single, commercially representative sourcing scenario within India, and results may vary under alternative sourcing or transportation assumptions. The impact of energy use (1.20–1.36 kg CO_2_ eq. per kg) represents a large hotspot for GWP; between 47% and 65% of total GWP impacts. Further decarbonisation strategies would involve engagement with policymakers within India, to facilitate the transition away from coal-powered energy and towards more renewable sources of energy.

Forfora *et al.*^21^ found that energy use, combined with CO_2_ fossil emissions from energy vectors, contributed towards 75% of GWP impacts of a paper-based product (jumbo roll), manufactured in China with a 70% bamboo and 30% hardwood fibre furnish. Manufacturing the same product in the USA reduced GWP from 2.05 kg CO_2_ eq. per kg to 1.90 kg CO_2_ eq. per kg, due to reduced coal dependence in the USA energy mix. Interestingly, Forfora *et al.*^21^ also reported a 10.4% lower GWP for jumbo roll produced using a virgin-wood fibre furnish (70% hardwood and 30% softwood), compared to the bamboo-containing product. In this case, bamboo pulp was imported from China, contributing 31% of total GWP impacts of the jumbo roll with 70% bamboo and 30% hardwood fibre furnish. The authors attribute this result to the coal-intensive energy mix used for manufacturing in China.

Raw material (fibre) sourcing contributes only 6% of total GWP impacts for BAU and 4–7% for papers containing bagasse, bamboo or OCC (Fig. 8A). Therefore, GWP impacts are largely a consequence of supply chain geography, energy mix and transportation routes, rather than fibre selection. This suggests that GWP benefits are driven by logistics rather than fibre properties and are likely location-dependent rather than universal. This is supported by Forfora *et al.*,^21^ who reported that biomass production contributed 18%, 14% and 6% towards total GWP impacts of hardwood, softwood and bamboo kraft pulps, respectively. Arfelli *et al.*^14^ also reported that biomass production minimally contributed to GWP impacts of paper-based confectionary packaging. Although biomass sourcing results in minimal GWP impact, Fig. S3 (SI) shows that fibre is the major contributing hot spot towards land use impacts (95%) and water consumption (63%) for the BAU scenario. Both land use and water consumption impacts are driven by biomass cultivation for softwood, hardwood, bagasse and bamboo and by the paper recycling process in the case of OCC. Utilising non-wood fibres consistently reduced land use change and water consumption impacts, compared to BAU. Replacing SW with bagasse, bamboo or OCC reduced land use change by 24%, 23% and 18% and water consumption by 28%, 25% and 42%, respectively, compared to BAU.

The contribution of combined additive inclusion to the environmental impacts is shown in Fig. 8B and S4 (SI). The GWP of the combined additives was 0.16 kg CO_2_ eq. per kg paper, representing 6–8% of total GWP impacts. CNFs account for the majority (95%) of additive-related GWP impacts due to its high production impact (6.0 kg CO_2_ eq. per kg CNF) driven by electricity demand. However, the overall impact is limited by low CNF loading into the paper (2.5%). This contribution will also depend on CNF production location and the energy-mix utilised by different geographic regions. Additives most significantly contribute towards ionising radiation (5–27%) and water consumption (8–15%) (Fig. S4, SI), whereas additives contribute minimally (<10%) to all other impact categories.

## Conclusions

This study demonstrates that incorporating locally sourced non-wood fibres (bagasse, bamboo and old corrugated cardboard) into paper production in India can provide environmental and economic advantages. Key hand sheet physical properties, including tensile index, porosity, air permeability and surface roughness, were maintained or improved. Substituting 20% of virgin wood fibres, in particular softwood fibres, reduces production costs by 11–12% and global warming potential by 27–29%, driven by reducing softwood imports, reducing transport impacts and establishing more localised supply chains. This reduction is specific to the sourcing scenario modelled, in which non-wood fibres displace imported softwood, and should not be interpreted as a universal advantage of non-wood fibres. Inclusion of combined additives, 2.5% cellulose nanofibrils (CNFs) and 1% cationic starch, demonstrates improvements in the tensile index, while reducing porosity and air permeability of the papers. Additives only contributed towards 5–6% of production costs and 6–8% of global warming potential impacts. Therefore, the combination of utilising locally sourced non-wood fibres in combination with CNF and cationic starch additives shows potential as a strategy to reduce environmental impacts and costs, while maintaining or improving key hand sheet physical properties. These results are encouraging for the potential use of these paper formulations in flexible packaging. However, additional treatments, such as barrier coatings, would likely be required to achieve necessary barrier properties, moisture resistance, wet strength, printability, grease resistance and sealability. Future research will aim to optimise hand sheet properties, cost and environmental impact by optimising fibre blend composition and additive inclusion levels. Further work will also explore supply chain optimisation, including alternative fibre and pulp sourcing locations and transport scenarios to better understand their influence on production costs and environmental impacts.

## Author contributions

AEB: conceptualisation, data curation, formal analysis, investigation, methodology, validation, visualisation, writing – original draft, writing – review & editing. PG: investigation, methodology. MS: data curation, formal analysis, investigation, methodology, validation. KI: data curation, formal analysis, investigation, methodology, validation. TT: methodology, resources, writing – review & editing. AR: methodology, resources. KYL: conceptualisation, methodology, resources, supervision, writing – review & editing. JB: conceptualisation, funding acquisition, project administration, resources, supervision, writing – review & editing. KW: conceptualisation, formal analysis, funding acquisition, methodology, project administration, resources, supervision, visualisation, writing – review & editing.

## Conflicts of interest

There are no conflicts to declare.

## Supplementary Material

SU-OLF-D6SU00291A-s001

## Data Availability

The supporting data have been provided as part of the supplementary information (SI). Supplementary information is available. See DOI: https://doi.org/10.1039/d6su00291a.
