# Supplementary material for: Technical feasibility, sustainability and technoeconomic analysis of non-wood fibre integration in paper-based flexible packaging: a case study of India
Source: RSC Sustain. 2026 Nov 9. Online ahead of print. doi: 10.1039/d6su00291a (PMC13746234; doi:10.1039/d6su00291a)
Supplement: Supplementary file 1 [file SU-OLF-D6SU00291A-s001.pdf]

Supplementary Information (SI) for RSC Sustainability.
This journal is © The Royal Society of Chemistry

Technical Feasibility, Sustainability and Technoeconomic Analysis of Non-Wood Fibres Integration in Paper-Based Flexible Packaging: A Case Study of India

Aaron E. Brown[1], Paulina Gordina[2], Meiju Sinkkonen[3], Karim Ismail[1], Tekla Tammelin[3], Andrew Ross[1], Koon-Yang Lee[2,4], John Blacker[5], Keeran Ward[1]*

[1] School of Chemical and Process Engineering, University of Leeds, Woodhouse Lane, Leeds LS2 9JT, UK

[2] Department of Aeronautics, Imperial College London, South Kensington Campus, London SW7 2AZ, United Kingdom

[3] VTT Technical Research Centre of Finland Ltd, VTT, PO Box 1000, FI-02044 Espoo, Finland

[4] Institute for Molecular Science and Engineering (IMSE), Imperial College London, SW7 2AZ, United Kingdom

[5] Institute of Process Research and Development, School of Chemistry, University of Leeds, Leeds LS2 9JT, UK

Corresponding author

Supplementary Information

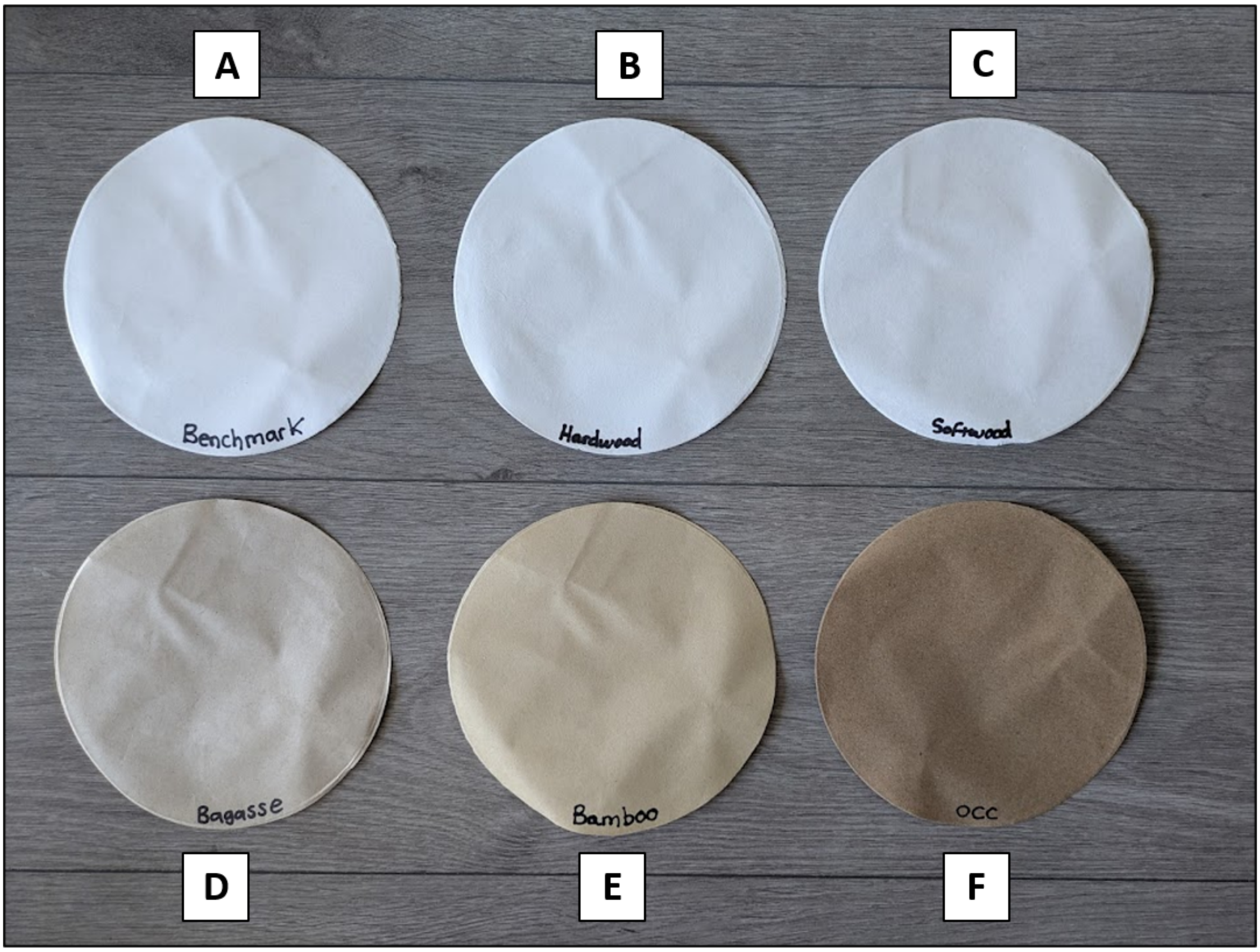

Figure S1. Images of produced hand sheets: (A) BAU, (B) HW, (C) SW, (D) BAG, (E) BAM, (F) OCC.

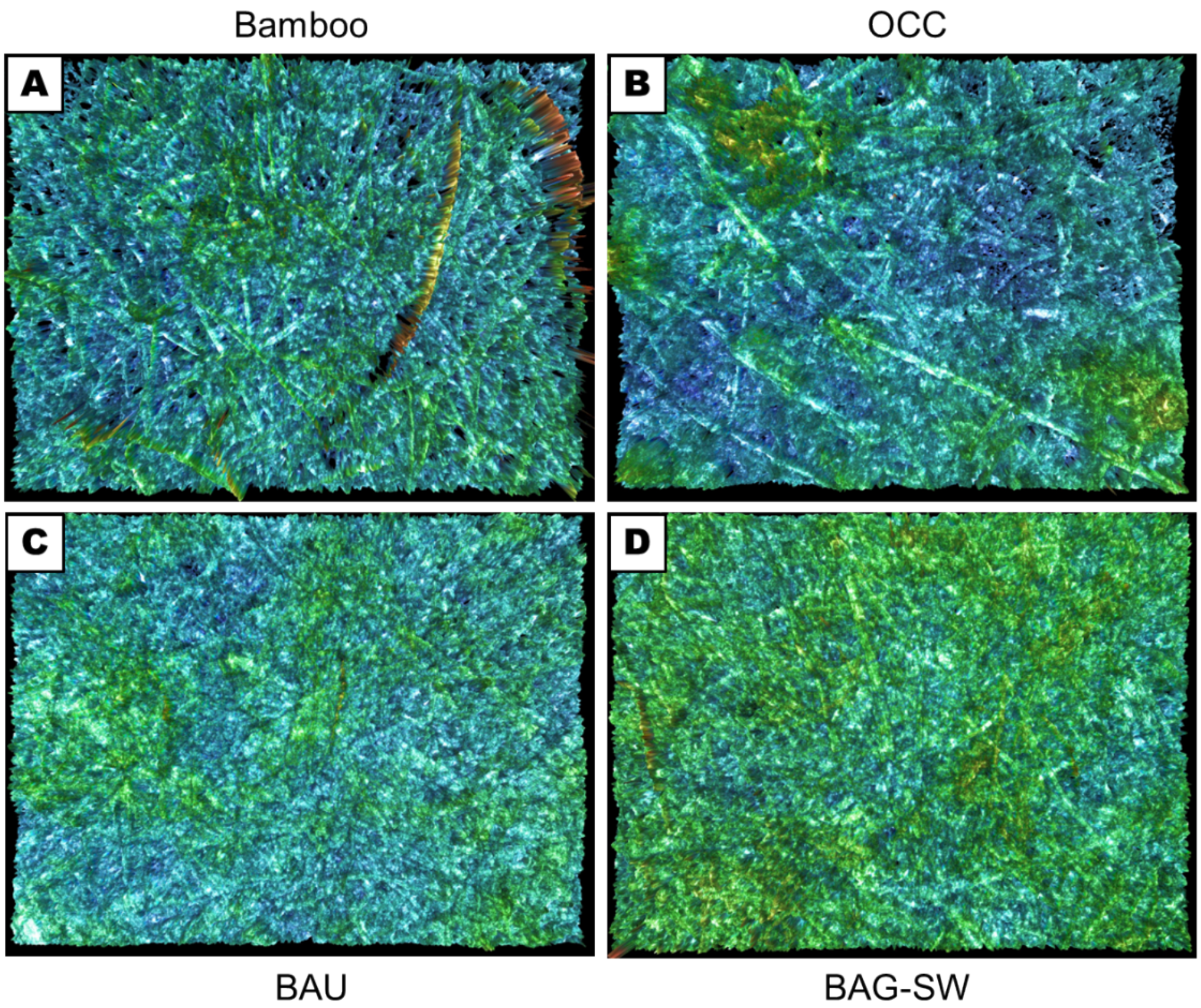

Figure S2. Confocal microscopy images depicting surface roughness of the hand sheets.

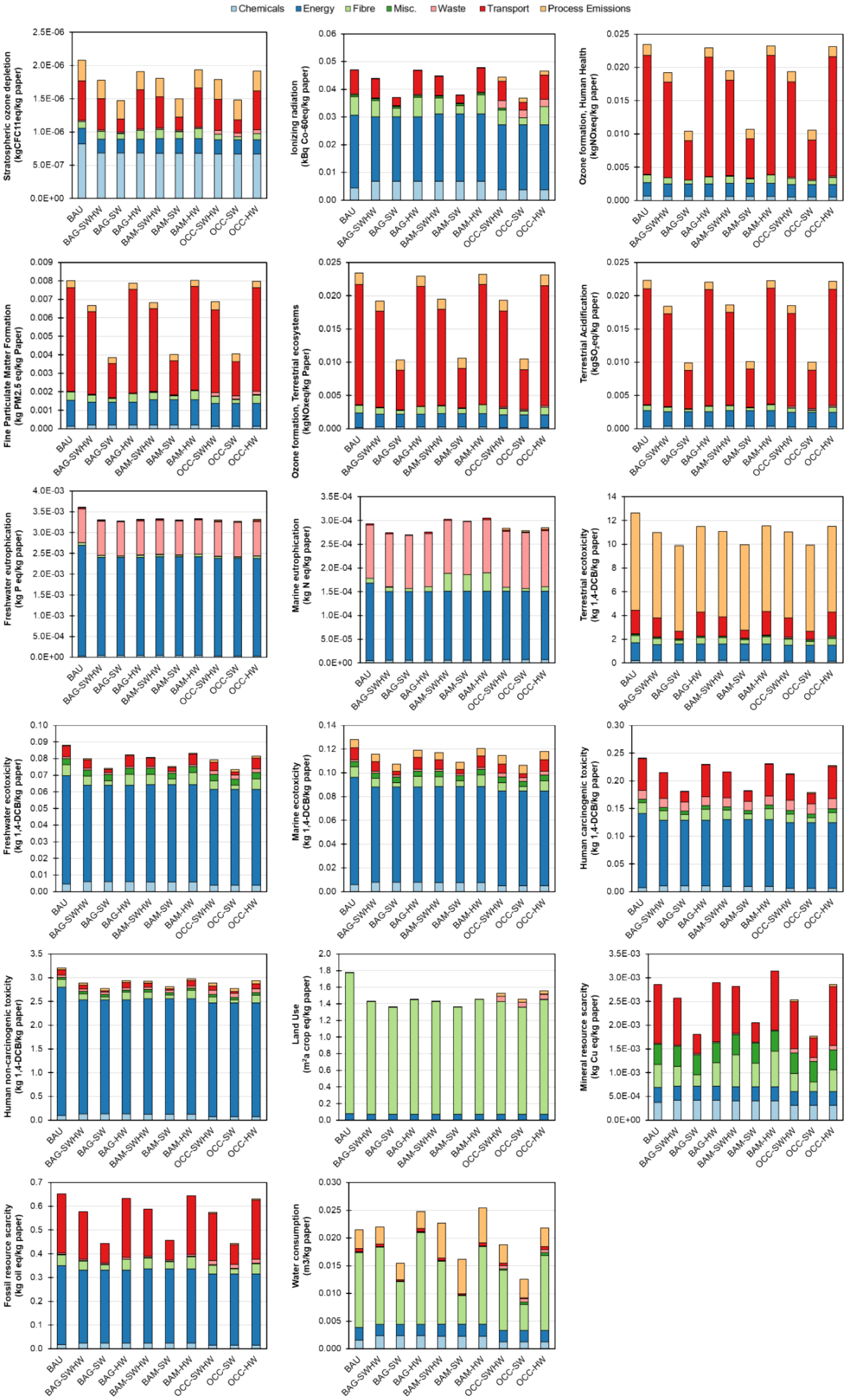

Figure S3. LCIA results across the additional 17 impact categories from ReCiPe (H) midpoint analysis (excluding global warming potential) for hand sheets produced without additives.

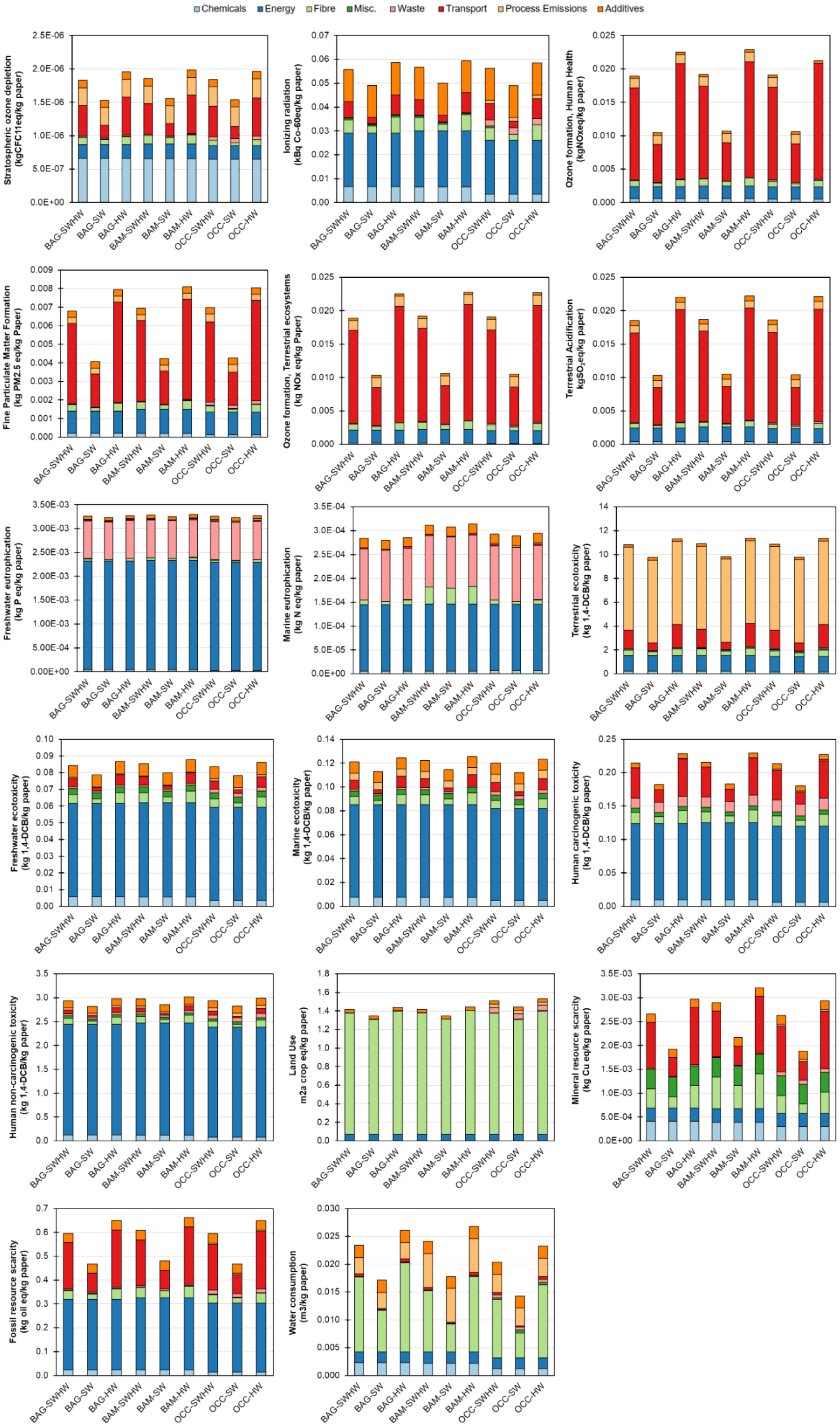

Figure S4. LCIA results across the additional 17 impact categories from ReCiPe (H) midpoint analysis (excluding global warming potential) for hand sheets produced with additives.

Table S1. Three-way ANOVA for Tensile Index and Porosity.

Source	df	Tensile Index		Porosity	
		F	*p*	*F*	*p*
Corrected Model	17	8.361	<0.001	17.953	<0.001
Fibre Furnish	2	18.586	<0.001	14.621	<0.001
Additive Inclusion	1	89.443	<0.001	261.241	<0.001
Substitution Strategy	2	0.209	0.812	0.432	0.652
Fibre Furnish x Additive Inclusion	2	4.297	0.021	3.809	0.032
Fibre Furnish x Substitution Strategy	4	0.356	0.838	0.342	0.848
Additive Inclusion x Substitution Strategy	2	0.558	0.577	0.974	0.387
Fibre Furnish x Additive Inclusion x Substitution Strategy	4	0.991	0.425	0.730	0.578
Error	36				
Corrected Total	53				

Design: 3 (Fibre Furnish: [BAG/BAM/OCC]) × 2 (Additive Inclusion: [Yes/No]) × 3 (Substitution Strategy: [SWHW/SW/HW]) full factorial, giving 18 formulations, each represented by three independent hand sheets (N=54). Model fit: Tensile Index, R^2=0.798 (adjusted R^2=0.702); Porosity, R^2=0.894 (adjusted R^2=0.845).

Table S2. Life cycle inventory for bagasse fibre.

Bagasse Fibre	Input/Output Amount	Inventory
**Raw Material**		
Nitrogen fertiliser/kg	3.65	inorganic nitrogen fertiliser, as N {RoW}| market for inorganic nitrogen fertiliser, as N | Cut-off, U
Phosphorus fertiliser/kg	12.16	inorganic phosphorus fertiliser, as P_2O_5 {RoW}| market for inorganic phosphorus fertiliser, as P_2O_5 | Cut-off, U
Potassium chloride/kg	13.68	potassium chloride {RoW}| market for potassium chloride | Cut-off, U
**Utilities**		
Water/kg	369,360	Tap water {RoW}| market for | Cut-off, U
Electricity/kWh	274.10	Electricity, high voltage {IN-Northern grid}| market for electricity, high voltage | Cut-off, U
Diesel/kg	34.23	diesel {IN}| market for diesel | Cut-off, U
**Emissions**		
VOC/kg	0.091	VOC, volatile organic compounds as C
Dinitrogen monoxide/kg	0.300	Dinitrogen monoxide
Nitrogen oxides/kg	0.631	Nitrogen oxides
Ammonia/kg	0.150	Ammonia
Carbon dioxide/kg	4.840	Carbon dioxide, fossil
Sulphur dioxide/kg	0.010	Sulphur dioxide
Nitrate/kg	1.060	Nitrate
Phosphate/kg	0.150	Phosphate
**Products**		
Bagasse/ton	1.00	-
Pith/ton	0.67	-
Sugar/ton	5.40	-
Molasses/ton	2.10	-

Inventory adapted from [1,2].

Economic allocation of impacts: Bagasse (1.3%), Pith (0.0%), Sugar (5.9%) and Molasses (92.7%).

Table S3. Life cycle inventory for bagasse pulp/paper.

Bagasse Pulp/Paper	Input/Output Amount	Inventory
**Raw Material**		
Bagasse fibre/kg	3236	Bagasse Fibre (Table S2)
Hydrogen peroxide/kg	7.4	Hydrogen peroxide, without water, in 50% solution state {RoW}| market for hydrogen peroxide, without water, in 50% solution state | Cut-off, U
Sodium chlorate/kg	21	Sodium chlorate, powder {RoW}| market for sodium chlorate, powder | Cut-off, U
Methanol/kg	2.2	Methanol {GLO}| market for | Cut-off, U
Sodium hydroxide/kg	57.8	Sodium hydroxide, without water, in 50% solution state {GLO}| market for | Cut-off, U
Sodium sulfate/kg	13.9	Sodium sulfate, anhydrite {RoW}| market for | Cut-off, U
Sulfuric acid/kg	12	Sulfuric acid {RoW}| market for sulfuric acid | Cut-off, U
Oxygen/kg	23.8	Oxygen
**Utilities**		
Biogas/m^3	12.9	Biogas {RoW}| market for biogas | Cut-off, U
Natural gas/m^3	25.6	Natural gas, high pressure {RoW}| market for | Cut-off, U
Fuel oil/kg	18.8	heavy fuel oil {IN}| market for heavy fuel oil | Cut-off, U
Coal/kg	118	Hard coal {IN}| market for hard coal | Cut-off, U
**Emissions**		
Carbon dioxide/kg	448.8	Carbon dioxide, fossil
Methane/g	24.3	Methane
Nitrogen dioxide/g	18.8	Nitrogen dioxide, IN
Sulfur dioxide/kg	4.53	Sulfur dioxide, IN
AOX/kg	0.3	AOX, Adsorbable Organic Halogen as Cl
Suspended solids/kg	4.3	Suspended solids, unspecified
Organic compounds/kg	27.6	Organic compounds (dissolved)
BOD/kg	4.5	BOD5 (Biological Oxygen Demand), IN

**Wastes**		
Waste/kg	34.3	Waste, final, inert
Dust/kg	0.5	Dust, unspecified
**Products**		
Bagasse Pulp/ton	1.00	-

Table S4. Life cycle inventory for bamboo fibre.

Bamboo Fibre	Input/Output Amount	Inventory
**Raw Material**		
Nitrogen fertiliser/kg	4.35	inorganic nitrogen fertiliser, as N {IN}| market for inorganic nitrogen fertiliser, as N | Cut-off, U
Phosphorus fertiliser/kg	1.70	inorganic phosphorus fertiliser, as P2O5 {IN}| market for inorganic phosphorus fertiliser, as P2O5 | Cut-off, U
Potassium fertiliser/kg	2.00	inorganic potassium fertiliser, as K2O {IN}| market for inorganic potassium fertiliser, as K2O | Cut-off, U
**Utilities**		
Power sawing/hr	0.87	Power sawing, without catalytic converter {RoW}| processing | Cut-off, U
Diesel/kg	3.22	diesel {IN}| market for diesel | Cut-off, U
**Emissions**		
Nitrogen oxides/kg	0.002	Nitrogen oxides
Nitrogen monoxide/kg	0.13	Nitrogen monoxide
Carbon dioxide/kg	0.46	Carbon dioxide
Sulphur dioxide/kg	0.001	Sulphur dioxide
VOC/kg	0.009	VOC, volatile organic compounds as C
Ammonia/kg	0.066	Ammonia
Phosphate/kg	0.003	Phosphate
Nitrate/kg	0.590	Nitrate
**Products**		
Bamboo Fibre/ton	1.00	-
Bamboo Cultivation Waste/ton	0.42	-

Inventory adapted from [3].

Allocation of impacts: Bamboo Fibre (100%), Bamboo Cultivation Waste (0%).

Table S5. Life cycle inventory for bamboo pulp/paper.

Bamboo Pulp/Paper	Input/Output Amount	Inventory
**Raw Material**		
Bamboo fibre/kg	2440	Bamboo Fibre (Table S4)
Sodium hydroxide/kg	54.8	Sodium hydroxide, without water, in 50% solution state {GLO}| market for | Cut-off, U
Sodium sulfate/kg	2.5	Sodium sulfate, anhydrite {RoW}| market for | Cut-off, U
Sulfuric acid/kg	15.3	Sulfuric acid {RoW}| market for sulfuric acid | Cut-off, U
Quicklime/kg	19.6	Quicklime, in pieces, loose {RoW}| production | Cut-off, U
Hydrogen peroxide/kg	3.6	Hydrogen peroxide, without water, in 50% solution state {RoW}| market for hydrogen peroxide, without water, in 50% solution state | Cut-off, U
Sodium chlorate/kg	21	Sodium chlorate, powder {RoW}| market for sodium chlorate, powder | Cut-off, U
Methanol/kg	2.2	Methanol {GLO}| market for | Cut-off, U
Oxygen/kg	35.8	Oxygen
Water/m^3	27.34	Water, unspecified natural origin, IN
**Utilities**		
Electricity/kWh	386.6	Electricity, high voltage {IN-Northern grid}| market for electricity, high voltage | Cut-off, U
Natural gas/m^3	22.1	Natural gas, high pressure {RoW}| market for | Cut-off, U
Fuel oil/kg	21	heavy fuel oil {IN}| market for heavy fuel oil | Cut-off, U
Coal/kg	8.3	Hard coal {IN}| market for hard coal | Cut-off, U
**Emissions**		
Carbon dioxide/kg	133.6	Carbon dioxide, fossil
Methane/g	180.6	Methane
Nitrogen dioxide/g	39.6	Nitrogen dioxide, IN
Sulfur dioxide/kg	0.2	Sulfur dioxide, IN
AOX/kg	0.8	AOX, Adsorbable Organic Halogen as Cl

BOD/kg	4.5	BOD5 (Biological Oxygen Demand), IN
**Wastes**		
Waste/kg	12.7	Waste, final, inert
Slags and ashes/kg	0.5	Slags and ashes
**Products**		
Bamboo Pulp/ton	1.00	-

Table S6. Life cycle inventory for OCC pulp/paper.

OCC	Input/Output Amount	Inventory
**Raw Material**		
Water/kg	400	tap water {IN}| market for tap water | Cut-off, U
Borax/kg	0.24	Borax, anhydrous, powder {GLO}| market for | Cut-off, U
Sodium hydroxide/kg	2.34	Sodium hydroxide, without water, in 50% solution state {GLO}| market for | Cut-off, U
Starch/kg	11.15	Maize starch {RoW}| production | Cut-off, U
**Utilities**		
Heat/GJ	0.032	Heat, from steam, in chemical industry {RoW}| market for heat, from steam, in chemical industry | Cut-off, U
Electricity/GJ	0.444	Electricity, high voltage {IN-Northern grid}| market for electricity, high voltage | Cut-off, U
Natural gas/GJ	0.803	Natural gas, burned in gas motor, for storage {GLO}| market for | Cut-off, U
Heavy fuel oil/kg	0.239	heavy fuel oil {IN}| market for heavy fuel oil | Cut-off, U
Light fuel oil/kg	0.2045	light fuel oil {IN}| market for light fuel oil | Cut-off, U
Diesel/kg	0.02193	diesel {IN}| market for diesel | Cut-off, U
Petroleum gas/kg	0.223	liquefied petroleum gas {IN}| market for liquefied petroleum gas | Cut-off, U
Coal/kg	0.448	Hard coal {IN}| market for hard coal | Cut-off, U
Wood/m^3	0.74	residual wood, dry {RoW}| market for residual wood, dry | Cut-off, U
**Emissions**		
Particulates, < 2.5 um/kg	0.00077	Particulates, < 2.5 um
Particulates, > 2.5 um, and < 10um/kg	0.0003	Particulates, > 2.5 um, and < 10um
Particulates, < 10 um/kg	0.00023	Particulates, < 10 um
Carbon dioxide/kg	47.62	Carbon dioxide
Nitrogen dioxide/kg	0.018	Nitrogen dioxide, IN

Sulfur dioxide/kg	0.011	Sulfur dioxide, IN
COD/kg	0.31	COD (Chemical Oxygen Demand), IN
Suspended solids/kg	0.074	Suspended solids, unspecified
Nitrogen/kg	0.0061	Nitrogen, total
Phosphorus/kg	0.0012	Phosphorus, total, IN
**Products**		
OCC/ton	1.00	-

Inventory adapted from [4].

Table S7. Life cycle inventory for business-as-usual paper production

BAU	Input/Output Amount	Inventory
**Raw Material**		
Water/m³	0.0559	Water, unspecified natural origin, IN
Aluminium sulfate/kg	0.00645	Aluminium sulfate, powder {RoW}| market for aluminium sulfate, powder | Cut-off, U
Carbon dioxide, liquid/kg	0.00112	Carbon dioxide, liquid {RoW}| market for | Cut-off, U
Chemical, organic/kg	0.000946	Chemical, organic {GLO}| market for | Cut-off, U
Hydrogen peroxide, kg	0.00103	Hydrogen peroxide, without water, in 50% solution state {RoW}| market for hydrogen peroxide, without water, in 50% solution state | Cut-off, U
Magnesium sulfate, kg	0.000258	Magnesium sulfate {GLO}| market for | Cut-off, U
Oxygen, liquid/kg	0.0293	Oxygen, liquid {RoW}| market for | Cut-off, U
Paper mill/p	4.68	Paper mill, integrated {GLO}| market for | Cut-off, U
Quicklime/kg	0.0293	Quicklime, milled, loose {RoW}| market for quicklime, milled, loose | Cut-off, U

Rosin/kg	0.000516	Rosin size, for paper production {RoW}| market for rosin size, for paper production | Cut-off, U
Soda ash/kg	0.00189	Soda ash, light, crystalline, heptahydrate {GLO}| market for | Cut-off, U
Sodium chlorate/kg	0.00482	Sodium chlorate, powder {RoW}| market for sodium chlorate, powder | Cut-off, U
Sodium hydroxide/kg	0.00100	Sodium hydroxide, without water, in 50% solution state {GLO}| market for | Cut-off, U
Sodium sulfate/kg	0.00284	Sodium sulfate, anhydrite {RoW}| market for | Cut-off, U
Sulfuric acid/kg	0.00671	Sulfuric acid {RoW}| market for sulfuric acid | Cut-off, U
Pulpwood, hardwood/m^3	0.00205	Pulpwood, hardwood, measured as solid wood under bark {RoW}| hardwood forestry, beech, sustainable forest management | Cut-off, U
Sulfate pulp, softwood/kg	0.300	Sulfate pulp, bleached {RoW}| sulfate pulp production, from softwood, bleached | Cut-off, U
Transport, sea ferry/tkm	7.70	transport, freight, sea, ferry {GLO}| transport, freight, sea, ferry | Cut-off, U
**Utilities**		
Heat, softwood/MJ	1.17	Heat, central or small-scale, other than natural gas {RoW}| heat production, softwood chips from forest, at furnace 50kW | Cut-off, U
Heat, fuel oil/MJ	1.05	Heat, central or small-scale, other than natural gas {RoW}| heat production, light fuel oil, at boiler 100kW

		condensing, non-modulating | Cut-off, U
Heat, lignite briquette/MJ	7.03	Heat, central or small-scale, other than natural gas {RoW}| heat production, lignite briquette, at stove 5-15kW | Cut-off, U
**Emissions**		
Acetaldehyde/kg	1.58E-07	
Acetic acid/kg	3.05E-07	
Acetone/kg	5E-08	
Aluminium/kg	7.61E-06	
Ammonia/kg	0.000253	
Antimony/kg	1.62E-08	
Arsenic/kg	3.85E-08	
Barium/kg	1.62E-07	
Benzene/kg	5.08E-06	
Benzene, ethyl-/kg	5.3E-08	
Benzene, hexachloro-/kg	1.27E-14	
Benzo(a)pyrene/kg	1.37E-08	
Beryllium/kg	1.62E-09	
Boron/kg	7.3E-06	
Bromine/kg	1.16E-07	
Butane/kg	5.05E-07	
Cadmium/kg	6.17E-09	
Calcium/kg	4.27E-05	
Carbon dioxide, biogenic/kg	2.24913	
Carbon dioxide, fossil/kg	0.130766	
Carbon monoxide, biogenic/kg	0.001015	

Carbon monoxide, fossil/kg	5.33E-05
Chlorine/kg	3.18E-07
Chromium/kg	5.35E-08
Chromium VI/kg	5.4E-10
Cobalt/kg	3.53E-08
Copper/kg	1.02E-07
Dinitrogen monoxide/kg	2.84E-05
Dioxin, 2,3,7,8 Tetrachlorodibenzo-p-/kg	5.63E-14
Ethane/kg	9.64E-06
Ethanol/kg	9.98E-08
Ethene/kg	1.92E-05
Ethyne/kg	3.2E-06
Fluorine/kg	8.86E-08
Hydrocarbons, aliphatic, alkanes, unspecified/kg	5.03E-06
Hydrocarbons, aliphatic, unsaturated/kg	8.69E-06
Hydrocarbons, aromatic/kg	5.18E-08
Hydrogen chloride/kg	2.61E-06
Hydrogen fluoride/kg	9.55E-08
Hydrogen sulfide/kg	2.49E-05
Iodine/kg	2.91E-08
Iron/kg	8.95E-06
Lead/kg	1.32E-08
Lead-210/kg	3.97E-05
Magnesium/kg	5.49E-06
Manganese/kg	3.17E-07

Mercury/kg	2.55E-09
Methane, biogenic/kg	5.3E-06
Methane, fossil/kg	7.48E-05
Molybdenum/kg	3.1E-08
m-Xylene/kg	2.12E-07
Nickel/kg	2.1E-07
Nitrogen oxides/kg	0.001429
NMVOC, non-methane volatile organic compounds, unspecified origin/kg	0.001055
PAH, polycyclic aromatic hydrocarbons/kg	2.68E-08
Particulates, < 2.5 um/kg	9.55E-05
Particulates, > 10 um/kg	9.03E-05
Particulates, > 2.5 um, and < 10um/kg	3.05E-05
Pentane/kg	8.46E-07
Phenol/kg	3.2E-07
Phenol, pentachloro-/kg	1.43E-11
Phosphorus/kg	5.95E-07
Polonium-210/kg	7.3E-05
Potassium/kg	4.23E-05
Potassium-40/kg	1.07E-05
Propane/kg	6.55E-06
Propene/kg	3.2E-06
Propionic acid/kg	1.39E-08
Radium-226/kg	1.02E-05
Radium-228/kg	5.51E-06
Radon-220/kg	2.56E-06

Radon-222/kg	2.56E-06	
Scandium/kg	4.04E-10	
Selenium/kg	5.25E-08	
Silicon/kg	7.74E-05	
Sodium/kg	4E-06	
Strontium/kg	1.69E-07	
Sulfur dioxide/kg	0.000241	
Thallium/kg	8.06E-11	
Thorium/kg	1.94E-09	
Thorium-228/kg	4.73E-06	
Thorium-232/kg	2.94E-06	
Tin/kg	1.29E-09	
Titanium/kg	1.62E-07	
Toluene/kg	1.32E-06	
Uranium/kg	3.23E-09	
Uranium-238/kg	8.58E-06	
Vanadium/kg	4.23E-05	
Water/m^3	0.003441	
Xylene/kg	2.11E-06	
Zinc/kg	5.96E-07	
AOX/kg	1.726E-05	AOX, Adsorbable Organic Halogen as Cl
BOD5/kg	0.000172	BOD5 (Biological Oxygen Demand)
Cadmium/kg	5.54E-09	Cadmium
Chloride/kg	0.000215	Chloride
Chromium/kg	7.41E-08	Chromium
COD/kg	0.00724	COD (Chemical Oxygen Demand)
Copper/kg	4.33E-07	Copper
DOC/kg	0.00236	DOC, Dissolved Organic Carbon
Lead/kg	1.05E-08	Lead
Mercury/kg	9.38E-11	Mercury

Nickel/kg	2.26E-08	Nickel
Nitrogen/kg	0.000224	Nitrogen, atmospheric
Phosphorus/kg	1.721E-05	Phosphorus
Suspended solids/kg	0.000912	Suspended solids, unspecified
TOC/kg	0.00237	TOC, Total Organic Carbon
Water/m^3	0.0525	Water, RER
Zinc/kg	1.13E-06	Zinc
Ash/kg	0.0245	Ash from paper production sludge {RoW}| market for ash from paper production sludge | Cut-off, U
Fly ash/kg	0.00779	Fly ash and scrubber sludge {RoW}| market for fly ash and scrubber sludge | Cut-off, U
Green liquor dregs/kg	0.00688	Green liquor dregs {GLO}| market for | Cut-off, U
Hazardous waste for incineration/kg	10.57	Hazardous waste, for incineration {RoW}| market for hazardous waste, for incineration | Cut-off, U
Hazardous waste for underground deposit/kg	8.60	hazardous waste, for underground deposit {RoW}| market for hazardous waste, for underground deposit | Cut-off, U
Limestone residue/kg	0.0399	Limestone residue {RoW}| market for limestone residue | Cut-off, U
Sludge/kg	0.0103	Sludge from pulp and paper production {RoW}| market for sludge from pulp and paper production | Cut-off, U
Waste wood/kg	0.0129	Waste wood, untreated {IN}| market for waste wood, untreated | Cut-off, U
**Products**		
BAU Paper/kg	1.00	-

Adapted from 'kraft paper {RER} | kraft paper production | cut-off, U' inventory.

Table S8. Life cycle inventory for CNF.

CNF	Input/Output Amount	Inventory
**Raw Material**		
Sulfate pulp/kg	1	Sulfate pulp, bleached {GLO}| market for | Cut-off, U
Water/kg	19.44	water, deionised {RoW}| market for water, deionised | Cut-off, U
**Utilities**		
Electricity/kWh	5.19	Electricity, low voltage {GLO}| market group for | Cut-off, U
Steam/MJ	7.11	Heat, from steam, in chemical industry {RoW}| market for heat, from steam, in chemical industry | Cut-off, U
**Output**		
CNF/kg	1	

Inventory adapted from [5]. CNF assumed to be produced through mechanical processes. Given the uncertainties in the CNF supply chain, a global electricity mix was applied, consistent with a global CNF supply chain assumption.

References

1 M. Munagala, Y. Shastri, K. Nalawade, K. Konde and S. Patil, *Waste Manag.*, 2021, **126**, 52–64.

2 T. Ramjeawon, *Int. J. life cycle Assess.*, 2004, **9**, 254–260.

3 J. Gan, M. Chen, K. Semple, X. Liu, C. Dai and Q. Tu, *Sci. Total Environ.*, 2022, **849**, 157937.

4 FEFCO, *European Database for Corrugated Board Life Cycle Studies 2025*, 2025.

5 S. Kane, S. A. Miller, K. E. Kurtis, J. P. Youngblood, E. N. Landis and W. J. Weiss, *Environ. Sci. Technol.*, 2023, **57**, 19137–19147.
